# Nanomaterials-Based Ion-Imprinted Electrochemical Sensors for Heavy Metal Ions Detection: A Review

**DOI:** 10.3390/bios12121096

**Published:** 2022-11-30

**Authors:** Liangyun Yu, Liangju Sun, Qi Zhang, Yawen Zhou, Jingjing Zhang, Bairen Yang, Baocai Xu, Qin Xu

**Affiliations:** 1 School of Light Industry, Beijing Technology and Business University, No. 11 Fucheng Road, Haidian District, Beijing 100048, China; 2 School of Environmental Science and Engineering, Yancheng Institute of Technology, Yancheng 224051, China; 3 College of Chemistry and Engineering, Yangzhou University, Yangzhou 225002, China

**Keywords:** electrochemical sensors, heavy metal ions, molecular imprinting, ion imprinting, nanomaterials

## Abstract

Heavy metal ions (HMIs) pose a serious threat to the environment and human body because they are toxic and non-biodegradable and widely exist in environmental ecosystems. It is necessary to develop a rapid, sensitive and convenient method for HMIs detection to provide a strong guarantee for ecology and human health. Ion-imprinted electrochemical sensors (IIECSs) based on nanomaterials have been regarded as an excellent technology because of the good selectivity, the advantages of fast detection speed, low cost, and portability. Electrode surfaces modified with nanomaterials can obtain excellent nano-effects, such as size effect, macroscopic quantum tunneling effect and surface effect, which greatly improve its surface area and conductivity, so as to improve the detection sensitivity and reduce the detection limit of the sensor. Hence, the present review focused on the fundamentals and the synthetic strategies of ion-imprinted polymers (IIPs) and IIECSs for HMIs detection, as well as the applications of various nanomaterials as modifiers and sensitizers in the construction of HMIIECSs and the influence on the sensing performance of the fabricated sensors. Finally, the potential challenges and outlook on the future development of the HMIIECSs technology were also highlighted. By means of the points presented in this review, we hope to provide some help in further developing the preparation methods of high-performance HMIIECSs and expanding their potential applications.

## 1. Introduction

Rapid industrial development and urbanization have brought about worldwide environmental pollution, including the almost ubiquitous contamination of heavy metal ions (HMIs), which are non-biodegradable and carry the risk of entering the human body through the food chain and poses a significant threat to human health when released into open areas [1,2]. The hazard extent of HMIs is closely related to their type, form, exposure time and dose, so their maximum allowable concentration in the environment is also different. Drinking water quality standards recommended by the World Health Organization (WHO), the European Union or the Environmental Protection Agency usually set maximum pollutant limits between the pm and ppb regimes [3]. Common toxic HMIs include Hg^2+^, Cd^2+^, Pb^2+^, Cu^2+^, Co^2+^, Cr^3+^, Mn^2+^, and so on. For example, Hg^2+^ can cause damage to kidneys, nervous system and heart system [4]. Pb^2+^ ingestion in the body can cause serious harm to the kidney, learning disabilities, decreased intelligence quotient (IQ), growth retardation and anemia [5]. Therefore, we need to develop inexpensive, simple, and sensitive methods for HMIs detection.

Currently, there are many kinds of conventional methods for detecting HMIs including surface-enhanced Raman spectroscopy (SERS), atomic absorption spectrometry (AAS), capillary electrophoresis (CE), inductively coupled plasma-atomic emission spectroscopy (ICP-AES), ion chromatography-ultraviolet vis spectrometry (IC-UV-vis), inductively coupled plasma mass spectrometry (ICP-MS), microprobe (MP), and X-ray fluorescence spectroscopy (XFS) [6,7]. However, these detection techniques have some drawbacks, such as high cost, time-consuming and complex instrument operation [8]. Due to the portability, low cost, reusability, good sensitivity, and high selectivity of ion-imprinted electrochemical sensors (IIECSs), which have overcome the limitations of the above methods, they have been developed for the HMIs detection in recent years [9]. It is the current research focus to further improve the selectivity of IIECSs and reduce the detection limit.

Molecular imprinting is an advanced technology that enables the preparation of molecularly imprinted polymers (MIPs) from tailored binding sites that complement the shape, size, and functional groups of template molecules [10]. The concept of “molecular imprinting” can be traced back to Polyakov’s research on imprinted silica gel for targeting a selective sorption of organic molecules in 1937 [11]. Then, being inspired by Linus Pauling’s theory of antibody formation, Dickey proposed to use a very similar method to prepare absorbents with specific recognition sites for dye molecules in silica gel in 1949 [12]. In 1972, Wulff and Sarhan prepared the first MIP based on covalent interaction for theophylline molecular separation [13]. MIPs have the merits including high temperature and pressure resistance, acid-base resistance property, good stability in organic solvents, strong reproducibility and regenerative ability and controllable film thickness, which makes them have great potential as sensor sensing elements [14,15]. Hence, MIPs have played a prominent role as an analytical tool in the development of custom sensors. Molecularly imprinted electrochemical sensors (MIECSs) combining the advantages of molecular imprinting technique (MIT) and electrochemical analysis were born [16]. MIECSs have attracted extensive attention owing to their specific recognition, rapidity, simplicity, and low cost, and have been successfully used to detect various molecules ranging from dangerous small molecules to biological macromolecules [17,18]. Ion imprinting technique (IIT) is a new technology emerging on the basis of MIT, and the materials produced using this technique are called ion-imprinted polymers (IIPs) [19]. As a branch of MIT, IIT has been regarded as a promising method for synthesizing materials owing to its capacity for realizing ion recognition with high specificity and selectivity [20]. IIPs differ from MIPs by altering the organic template molecules to ions [21]. The imprinting process is performed by copolymerization of functional monomers and a crosslinker in the presence of templates [22]. There are differences in the choice of functional monomers between MIPs and IIPs. For MIPs, they depend on the molecular structure of the template, and the common functional monomers include methacrylic acid (MAA), methyl methacrylate, vinylphenol, pyrrole(Py), toluene, resorcinol, o-phenylenediamine (OPD), phenol, etc. [23]. However, for IIPs, the functional monomers including MAA, acrylic acid (AA), acrylamide (AM), 4-vinylpyridine (4-VP), etc., are divided into two categories that one with double bonds, and the other with functional groups such as amino groups and hydroxyl groups, which can chelate with metal ions [24]. IIPs have some benefits similar to those of MIPs, including high chemical stability, ease of preparation, low cost, predetermined recognition ability, repeatability, and adaptability to harsh environment, so as to improve the selectivity and sensitivity of electrochemical sensors (ECSs) [25]. IIPs have special coordination or electrostatic interaction functions compared to most MIPs and are usually compatible with aqueous media [26]. Moreover, the memory effect generated during the preparation of IIPs has ensured the recognition of the target metal ions with high selectivity [21,27]. In a word, combining the advantages of electrochemical sensors such as high sensitivity, low cost and easy miniaturization, IIECSs constructed with IIPs as the sensitive material of electrochemical sensor not only has the advantages of simple preparation process and easy operation, but also has better selectivity, sensitivity and stability than the conventional ECSs [19].

Today, the unparalleled achievements of nanotechnology have given researchers a great opportunity to apply nanomaterials to the preparation of electrodes to obtain ultra-sensitive surfaces with higher selectivity [28]. Nanomaterials are classified as zero-, one-, two-, and three-dimensional nanostructures that exist as single, fused, aggregated, or spherical, tubular, and irregularly shapes [29]. They show characteristics of unique and remarkable electrical conductivity, strong electrocatalytic activity, favorable mechanical properties, good stability, high surface energy and easy functionalization [15]. The charge transfer of imprinted polymer on the electrode surface can be effectively improved by using nanomaterials functionalized imprinted electrode and the synthesized novel nanomaterials can fine-tune the selectivity and sensitivity of the electrode system [18,30,31]. The nanomaterial-improved sensors also exhibit size dependence and high functionalization and the sensitivity and selectivity can be significantly improved by anchoring of organic ligands to the surfaces, formation of nanocomposites, and covalent functionalization of bare electrodes [6]. Although IIPs have many advantages, they also have some disadvantages such as poor electrical conductivity and electrocatalytic activity, so it is very necessary to improve the low sensitivity of the corresponding sensor by using various nanomaterials. Heavy metal ions imprinted electrochemical sensors (HMIIECSs) prepared from nanomaterial-modified electrodes have been widely utilized in the field of HMIs detection. Hence, the present review aimed to briefly narrate the fundamentals and current preparation methods of nanomaterials-based HMIIECSs for HMIs detection, especially those prepared by carbon-based nanomaterials (such as carbon nanotubes, graphene and graphitic carbonitride nanomaterials), noble-metal nanomaterials (such as gold and silver nanomaterials), and metal oxide nanomaterials (such as zinc oxide), magnetic iron oxide nanomaterials, and silica nanomaterials. The challenges or future development directions of these HMIIECSs in the research area were also prospected. By means of the points presented in this review, we hope to provide some help in further developing the preparation methods of high-performance HMIIECSs and expanding their potential applications.

## 2. Preparation of IIPs

### 2.1. Preparation Principle of IIPs

**Figure 1:**
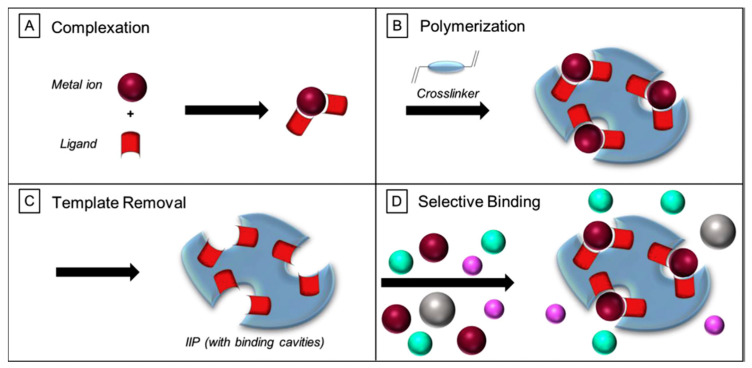
Schematic diagram of synthesis principle of IIP: (**A**) Complexation, (**B**) Polymerization, (**C**) Template Removal, (**D**) Selective Binding. Reprinted with permission from Ref. [24]. Copyright 2022, Elsevier.

IIT is an efficient and promising strategy for synthesizing functional monomers and crosslinkers into artificially selective recognition materials (IIPs) in the presence of target template ions through coordination, electrostatic and other actions [25]. The template ions are first bonded to functional monomers in the form of covalent, semi-covalent or non-covalent binding, and then the combined units are chemically fixed by the polymerization of crosslinkers to enhance the stability and mechanical properties of the composite polymer loading templates [11]. After crosslinking polymerization, the template ions are removed by elution with acid and distilled water to obtain rigid polymers with specific arrangement groups, fixed hole size and shape, and three-dimensional holes with specific selectivity for target ions are formed [32]. The preparation principle was shown in Figure 1. IIPs not only retain the advantages of MIPs, such as structure predetermination, specific recognition, wide practicability, and excellent corrosion resistance, but also have the function of strong recognition of imprinted ions, which provides a new research direction for the detection of trace HMIs [33,34].

### 2.2. Preparation Methods of IIPs

Over the years, in order to improve the sensitivity, stability, adsorption and other properties of IIPs, a great variety of strategies for imprinting methods, such as bulk, precipitation, suspension and emulsion polymerization, and surface imprinting technique, have extensively been developed [35].

#### 2.2.1. Bulk Polymerization

Bulk polymerization is a method in which monomers, crosslinkers, initiators and templates are mixed in a vessel to undergo an exothermic polymerization reaction, and then the resulting material is milled and screened to obtain the desired particle size range [36,37]. It is one of the most widely used methods because of its simplicity, ease of use, and the need for no complicated instrumentation [38]. The first IIP was made through bulk polymerization process using a mixture of monomer, initiator, crosslinker, and template [39]. In spite of this, it does have several important drawbacks such as the following: (1) The process of mechanical grinding and sieving takes a lot of time and the produced particles are not uniform in size and shape; (2) Grinding may be detrimental to some action sites, which greatly reduces the number of effective imprinted sites; and (3) Many of the binding sites in the polymer matrix are buried too deep to be reached even after repeated cleaning [40].

#### 2.2.2. Precipitation Polymerization

Precipitation polymerization is a heterogeneous polymerization method, which refers to the method where the generated polymer is insoluble in its monomer, or the monomer and initiator are soluble in the reaction medium, while the generated polymer is insoluble in the reaction medium, and the polymer is precipitated from the reaction system after formation [21]. The size and shape of the synthesized polymer particles depend on the ratio of monomer to solvent, and their particle sizes usually range from 0.3 to 10 μM [21]. Compared with bulk polymerization, precipitation polymerization has a higher yield, but the use of solvent is larger and the polymerization reaction time is longer [21]. Chen et al. developed a convenient method for sensitive and selective determination of Ce^3+^ in aqueous phase based on a carbon paste electrode (CPE) modified with IIPs synthesized by precipitation polymerization using Ce^3+^ as template, allyl phenoxyacetate (APA) as functional monomer, ethylene glycol dimethacrylate (EGDMA) as crosslinking agent, azobisisobutyronitrile (AIBN) as initiator, and methanol as porogen [41].

#### 2.2.3. Suspension Polymerization

In suspension polymerization, monomers are dispersed into monomer droplets in the dispersed phase containing water, monomer, initiator and porogen, and suspended in water under the action of agitation and dispersant [19]. The monomer droplet is equivalent to a small reactor for bulk polymerization, and its polymerization mechanism is the same as that of bulk polymerization [42]. Compared with the bulk polymerization method, the adsorption capacity, affinity constant and homogeneity of the associated binding sites of the IIPs prepared using this method were significantly improved [43]. Suspension polymerization usually results in large spherical polymer beads in the range of micron to millimeter, which is very conducive to the large-scale synthesis of imprinted polymers. It is very important to choose the appropriate dispersion medium because it may adversely affect the identification of targets [37]. Chaipuang et al. prepared copper(II) IIPs by suspension polymerization using copper(II) sulfate pentahydrate as the template ion, 4-VP and MAA as the functional monomers, EGDMA as the crosslinker, 1,1′-azobis(cyclohexanecarbonitrile) as the initiator, toluene as the porogen, and hydroxypropyl methyl cellulose as the stabilizer in the polymerization reaction [44]. Mishra and Verma synthesized ion imprinted and carbon nanofibers-grafted polymeric (CNF-IIPs) beads by suspension polymerization using Pb^2+^ as the template, allyl thiourea as the ligand, CNF as the carrier material, EGDMA as the crosslinking agent, toluene as the porogen, and AIBN as the initiator [45].

#### 2.2.4. Emulsion Polymerization

Emulsion polymerization is a heterogeneous polymerization method, that is, the monomer droplets with surfactant are emulsified and polymerized in a continuous phase containing initiator, and then small micelles are formed usually about 0.1–1 μm in diameter and stable polymer particles are also formed in the aqueous phase [39]. This method has the advantages of easy leaching of template ions and large, regular and uniform polymer particle size. He et al. synthesized a novel Ni (II) IIPs based on magnetic multi-walled carbon nanotubes (Fe_3_O_4_/MWCNTs-COOH) by inverse emulsion polymerization with chitosan (CS) and AA as the functional monomers, Ni(II) as the template, and N, N’-methylene double acrylamide (NNMBA) as the crosslinker [46].

#### 2.2.5. Surface Imprinting Method

Surface imprinting method is to confine the binding site to the surface of solid phase support with good accessibility by surface modification or coating and then crosslinking polymerized [36]. The technique is simple and environmentally friendly, and the obtained IIP particles are small and moreover easy to combine and separate with template ions quickly, exhibiting a high mass transfer rate and rapid binding kinetics [45,47]. In recent years, the surface imprinting method has been used as an alternative to other types of polymerization become a highly using IIPs preparation technique [48]. Ismail et al. synthesized a graphite-based IIP (G-IIP) which exhibits a potential adsorbent for wastewater treatment by using the surface-printing technique with allylthiourea as the functional monomer, nitrate as the template, and EGDMA as the crosslinker [49]. Fayazi et al. utilized novel magnetic Pb(II) ion-imprinted polymer (Pb(II)-MIIP) nanoparticles (NPs) synthesized by the surface imprinting technique with 4-VP (functional monomer), EGDMA (crosslinker), 2,3,5,6-tetra(2-pyridyl) pyrazine (TPPZ, chelating agent), and magnetic multi-walled carbon nanotubes (MMWCNTs, carrier) for the sensitive and selective detection of Pb(II) ions [50].

### 2.3. Preparation of HMIIECSs

#### 2.3.1. Preparation Principle of HMIIECSs

**Figure 2:**
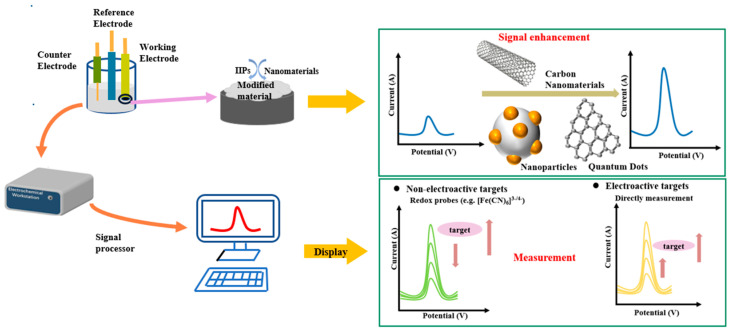
Construction and detection principle of HMIIECSs. Adapted with permission from Ref. [57]. Copyright 2021, Elsevier.

ECSs are a kind of device based on the electrochemical properties of the analytes, which convert their chemical amounts into electrical signals for sensing and detection [51]. They are a combination of electrical analysis technology and sensing technology. As an important means of analysis and detection, ECSs have aroused extensive interest in recent years due to its advantages such as high sensitivity, good selectivity, easy operation, strong anti-interference, suitable for miniaturization and real-time online detection [52]. ECS mainly consists of a recognition system and a conduction system [53]. The recognition system mainly selectively reacts with the measured substance and converts the resulting chemical signals into current, resistance, potential and other signals, while the conduction system accepts the signals and converts them into readable ones that can be analyzed by computers [54]. As is shown in Figure 2, a three-electrode system is generally used in the construction of ECSs, including working electrodes (WE), reference electrodes (RE) and counter electrodes (CE) [53]. The WE is very important since it influences the electrochemical sensitivity due to the redox reaction between the electrode material and the electrolyte [55]. Recently, various modified electrodes have become the focus of research since the surface functionalization of the WE can greatly improve the work efficiency, especially those modified with IIPs [6].

The detection principle of IIECSs is to combine IIT with an electrochemical detection method (Shown in Figure 2). The polymer prepared by the imprinting technology is used as the sensitive element and then modified to the electrode surface in various methods. When the IIPs bind to the ions in the solution, the electrochemical signal will change with the solution concentration. The response signals are finally detected and analyzed so as to detect the target ions. Generally, the selectivity of IIPs is used to determine HMIs based on the classical potentiometry and voltammetry. In the potentiometric method, IIPs replace the classical ionic groups used in ion selective electrodes (ISEs), and IIPs NPs-based HMI-ISEs have commenced a new era in the design of a new generation of potentiometric sensors, which have unique selectivity for the corresponding ions and a very low detection limit of 10^–8^–10^−10^ M [56]. Whereas in voltammetry, the role of IIPs is to selectively accumulate HMIs and these IIPs-modified electrodes exhibit much higher selectivity owing to their affinity for the specific size, charge, and coordination geometry of the target ions, and gain continued interest because of their high selectivity, low limit of detection (LOD), high accuracy, short analysis time, simple preparation and minimal sample pretreatment [6].

#### 2.3.2. Preparation Methods of HMIIECS

According to the different electrode modification methods, HMIIECS can be prepared by the direct and indirect methods [19].

##### The Direct Method

**Figure 3:**
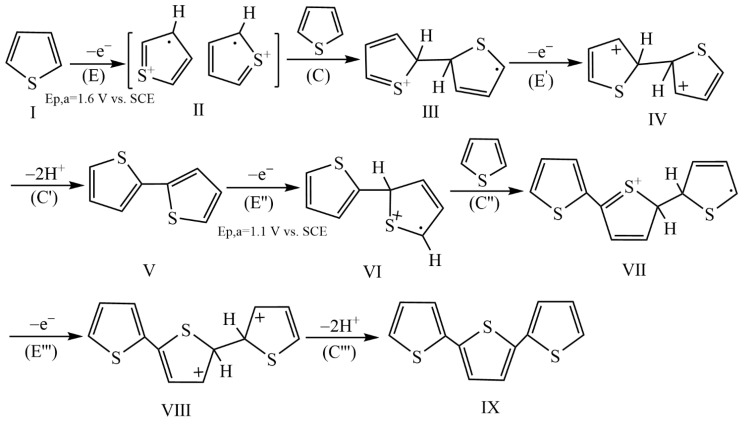
Basic mechanism of Th electropolymerization.

The direct method refers to that of forming IIPs directly on the electrode surface, which can be divided into in situ polymerization and electropolymerization [19,58]. In situ polymerization is a method of mixing functional monomers, template ions and initiators under certain conditions such as light or heat, and then applying them to the electrode surface for polymerization to form ion-imprinted membranes (IIMs). The thickness, hydrophobicity and permeability of IIPs prepared by this method can be artificially controlled and these IIPs are usually non-conductive, so conductive additives such as graphene or GO etc. are added to the pre-polymerized mixture [24]. The HMIIECSs prepared by this method presents simple and good recognition performance, but their IIMs are easy to fall off and their reproducibility is poor. Table 1 shows the comparison of this class of HMIIECSs. Electropolymerization means that the electrode is placed in a solution mixed with the functional monomers and the template ions in a certain proportion. Under the condition of applying electric potential or current, the functional monomers will polymerize on the electrode, and the template ions will be embedded in the polymeric film to form an IIM on the electrode surface. It is a good alternative approach, which can directly produce a uniform and selective film on the transducer surface and can overcome the problems associated with the fixation procedure [59]. Additionally, the synthesis of IIMs can be easily controlled by electrochemical methods to obtain the controllable amount of imprinted cavities. The mechanism of electropolymerization is still incompletely understood, nevertheless, the simplified one of the electroactive monomer, such as thiophene (Th) or Py involving alternate chemical (C) and electrode (E) reaction steps can be discussed [60]. As reported in this reference, taking Th electropotential polymerization as an example (shown in Figure 3), Th is firstly electrooxidized to form a free radical cationic II in step (E), which appears as an anode peak of high positive potential (Ep,a = 1.6 V vs. the saturated calomel electrode, SCE). In the second step (C) reaction, II reacts with the monomer I to form a protonic dimer of the free radical cation III. In the third step (E′) reaction, III is electrooxidized to the so-called double-charged σ-dimer IV, which cancels out the two protons to form a neutral dimer V at the following (C′) step. This dimer of V is then oxidized to form a free radical cation VI in the (E″) step. In turn, VI reacts with I in the (C″) step. The trimer IX is formed by these steps following the ECE′C′E″C″ mechanism in sequence. It is advantageous that the electrooxidation potential of the dimer V (Ep,a = 1.1 V vs. SCE) is lower than that of the monomer I. Therefore, many researchers are interested in this technique and have achieved good results in the water quality of HMIs detection. Di Masi et al. reported a sensitive and highly selective voltammetric sensor for the determination of copper(II) in water samples by electropolymerization of p-phenylenediamine (PPD) with Cu^2+^ as the template ions on the surface of screen-printed electrodes [59]. Cyclic voltammetry (CV), differential pulse voltammetry (DPV), scanning electron microscopy (SEM) analysis, and FTIR-ATR measurements were employed to characterize the proposed sensor. Good detection performance was obtained in acetate buffer (50 mM, pH = 5) that the linear range of Cu^2+^ was 0.95–244 nM with a LOD of 2.7 nM, and the analytical application in a spiked water sample revealed a good recovery of 95–105%. Table 2 lists the composition and main results of this kind of HMIIECSs.

##### The Indirect Method

In the indirect methods, IIPs are synthesized first by bulk, precipitation, or suspension polymerization and so on and eluted the template ions, and then immobilized on the electrode surface by means of a medium, which can be divided into a coating method (modifying IIPs onto the electrode surface by various coating methods) and combination method (mixing IIPs with electrode materials in a certain proportion and then modifying the electrode) [19]. In the coating method, a uniform IIM will be formed on the electrode surface after the solvent volatilizes. Chen et al. fabricated an Eu(III) ion-imprinted sensor on a screen printed electrode (SPE) (Eu(III)-IIM/PC/SPE) by using a poly catechol (PC) film as a signal amplifying element and an Eu(III) IIM as the recognition material [72]. In this work, the Eu(III) IIM was prepared via dripping the imprinted sol on the surface of PC/SPE, drying to be solidified under the ambient temperature, and then eluting Eu(III) in HCl. The prepared HMIIECS based on the coating method has good recognition, but the thickness of IIM could not be controlled, and long response time was required for detection. By contrast, the combination method is widely used in the field of analysis and detection, among which the most classical way is the modified CPE. This kind of HMIIECSs has the advantages of cheap materials, low residual current and easy surface renewal, but also has some shortcomings such as poor accuracy and poor reproducibility. Fadillahn et al. prepared IIP-CPE for voltammetry detection of Hg^2+^ by the combination method [73]. In detail, the Hg^2+^ IIP was synthesized by thermal bulk polymerization with Hg(NO_3_)_2_ as the template, 1,5-dipenylcarbazone (DPC) as the ligand, and EGDMA as the monomer. Then, the imprinted graphite powder with the oil wax was mixed at a ratio of 7:3 (weight ratio), and the formed carbon paste was inserted into a cylindrical electrode holder glass (d = 3 mm) and contacted with a copper rod to obtain IIP-CPE. Rebolledo-Perales et al. synthesized the Hg-IIP by using Py as the functional monomer, methanol as the porogen, EGDMA and sodium persulfate as the crosslinker and the initiator, respectively and constructed the modified electrode (CPE-IIP) by packing a composite mixture of 0.02 g of IIP, 0.18 g of graphite powder, and 0.08 g of paraffin oil into a plastic tube (0.50 mm in diameter and 25 mm in depth) situated at one end of the tube [32]. The obtained sensor presented a linear range of 0.09 to 20.05 mg/L with a LOD of 0.02 mg/L. The sensor successfully determined Hg(II) in three spiked samples (10 mg/L) from natural water sources, and the recovery rate was close to 99.74%, indicating that it can be directly applied to the detection of these samples.

## 3. Application of Nanomaterials in HMIECSs

In the electrochemical system for detecting HMIs, nanomaterials such as graphene oxide, carbon nanotubes and carbon nanofibers are considered to play an important role in improving the sensitivity of electrode sensors due to their good electrical conductivity, large effective activity and specific surface area [74]. This section discusses the development of different types of nanomaterials from the perspective of materials, focusing on the application research of nanomaterials in the field of HMIs imprinting electrochemical analysis, including carbon-based nanomaterials (such as carbon nanotubes, graphene, graphene oxide, reduced graphene oxide) and carbon-nitrogen nanomaterials), noble-metal nanomaterials (such as gold and silver nanomaterials), metal oxide nanomaterials (such as zinc oxide and magnetic iron oxide nanomaterials), silica nanomaterials, and so on.

### 3.1. Carbon-Based Nanomaterials

#### 3.1.1. Carbon Nanotube Materials

**Figure 4:**
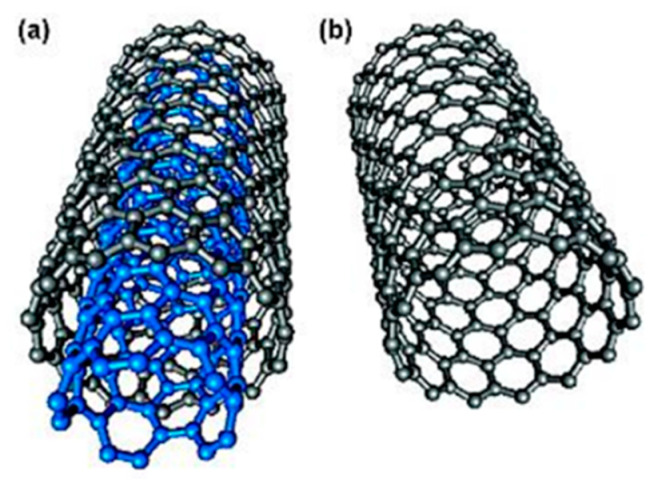
The structures of (**a**) MWCNTs and (**b**) SWCNTs. Reprinted with permission from Ref. [80]. Copyright 2009, the American Chemical Society.

Carbon nanotubes (CNTs) are composed of two defined structural groups, single-walled carbon nanotubes (SWCNTs) and MWCNTs as shown in Figure 4 [75,76]. CNTs are sp^2^ hybridization based on carbon atoms, which not only have unique electrical properties and low resistance, but also have significant stiffness and large axial strength, high active center specific surface area, controllable pore size distribution and excellent adsorption performance [77]. They can form complexes with HMIs, making it suitable for the development of electroanalytical systems specially used for the detection of HMIs [78]. Moreover, CNTs have higher electronic conductivity in electron transfer reactions and better electrochemical and chemical stability in aqueous and non-aqueous solutions, so they are suitable for the modification of various electrodes [79].

Rajabi et al. reported a voltammetric sensor with a novel IIP NPs and MWCNTs-modified glassy carbon electrode (GCE-IIP-MWCNTs) for selective recognition and sensitive determination of mercury ions by DPV [81]. The Hg^2+^ IIP nanobeads were prepared by precipitation polymerization, in which 5,10,15,20-tetrakis(3-hydroxyphenyl)-porphyrin (THPP) was added into the mixture of acetonitrile/dimethylsulfoxide as the porogen solvent, MAA as the functional monomer, EGDMA as the crosslinker monomer, and Hg^2+^ as the template. The IIP NPs in MWCNT/GCE acted as both selective inducer and preconcentrator, making the sensor have higher selectivity and lower LOD. The designed electrochemical sensor had a wide linear response range of 1 × 10^−8^–7.0 × 10^−4^ M and a LOD of 5.0 nM.

Nasiri-Majd et al. synthesized a simple and selective thallium-imprinted polymer (Tl-IP) by bulk polymerization with EGDMA as the crosslinking monomer, MAA as the functional monomer and AIBN as the initiator, and utilized the CPE modified with nano-sized Tl-IP and CNTs to fabricate the electrochemical sensor for the selective preconcentration and determination of Tl(I) by differential pulse anodic stripping voltammetry (DPASV) [82]. Under the optimized conditions, the suggested Tl-IP-MWCNT-CPE exhibited a wide concentration range of 3.0–240 ng/mL and low LOD of 0.76 ng/mL, respectively. In addition, it had good characteristics such as the simple preparation, reproducible surface renewal by simple polishing, excellent selectivity, high sensitivity and suitable stability and was successfully applied for the electrochemical determination of trace amounts of Tl(I) in water, hair and synthetic samples.

Alizadeh et al. prepared a voltammetric sensor for the determination of Cr^3+^ ion by modifying a CPE with Ce-IP, which was synthesized via precipitation polymerization using VP as the complexing ligands, MAA as the functional monomers, divinylbenzene (DVB) as the crosslinker and AIBN as the radical starter [83]. Then, the CPE was impregnated with Ce-IP and used for extraction and subsequent determination of Ce^3+^. The square wave voltammetry (SWV) showed that the response of this electrode was more significant than that of electrodes modified with non-IPs. The addition of MWCNTs to the Ce-IP modified electrode further improved the signal and increased the sensitivity of the method. Ce-IP-MWCNTs-CPE showed a linear response to Ce^3+^ in the concentration range of 1.0 to 25 pM with a LOD of 10 pM, and this method has been successfully applied to the determination of trace amounts of Ce^3+^ in drinking water and seawater. Again, Alizadeh et al. developed an inexpensive and simple potentiometric sensor based on CPE modified with IIP and MWCNTs to selectively recognize Cr^3+^ [84]. The all-solid-state Cr^3+^-selective sensor was obtained by using IIP as an ionophore. Adding an appropriate amount of MWCNTs to the electrode composition was found to be necessary for observing the Nernstian response. The optimized electrode composition was 76.7% graphite, 14.3% binder, 5% IIP, and 4% MWCNTs. The electrode response was significantly improved owing to the combination of MWCNTs with IIP. The proposed sensor displayed a Nernstian slope of 20.2 ± 0.2 mV decade^−1^ in the working concentration range of 1.0 × 10^−6^–1.0 × 10^−1^ M with a LOD of 5.9 × 10^−7^ M and a fast response time of less than 40 s. It showed a stable potential response in the pH range of 2–5 with high selectivity to some interfering ions, and has been successfully applied to the Cr^3+^ in real samples (seawater, river water and soil) measure.

Zhiani et al. synthesized a new nano-silver-IP, namely Ag-IIP-MWCNTs, for stripping voltammetric detection of Ag^+^ [85]. The electrochemical process was based on the accumulation of Ag^+^ ions on the surface of a nanosized silver IIP and MWCNTs modified CPE. After optimization, the Ag^+^ concentration showed a linear relationship between 0.5 × 10^−9^–2.8 × 10^−7^ M, and the LOD was 1.2 × 10^−10^ M (serial number = 3). The Relative standard deviation (RSD) of 9 consecutive measurements on different electrodes was 2.8%, and the interference study showed that several common metal ions did not interfere with the quantitative determination of Ag^+^. Due to the high selectivity and sensitivity of this system, it was applied to the determination of Ag^+^ in environmental water samples and standard materials.

Tarley et al. introduced the development of a highly selective Pb^2+^ ion electrochemical sensor based on a drop-coated GCE and an IIP suspension loaded with 1-(2-pyridazo)-2-naphthol (PAN) and MWCNTs [86]. Competitive adsorption studies on binary Pb^2+^/Cu^2+^, Pb^2+^/Cd^2+^, Pb^2+^/Ni^2+^, Pb^2+^/Ni^2+^, Pb^2+^/Zn^2+^ mixtures revealed their respective relative selectivity coefficients (*k*′) 301, 13.3, 9.5, 63.0 and 133.3, thus indicating that IIP-PAN had higher selectivity than NIP. The presence of potentially interfering metal ions was also investigated and demonstrated that the proposed electrode was very tolerant. In the range of 5.0–10.0 μg/L, the LOD and limit of quantification (LOQ) were 0.16 μg/L and 0.50 μg/L, respectively. The developed sensor was effectively applied in water samples and synthetic urine with recoveries ranging from 95% to 103%.

Alizadeh et al. prepared Pb^2+^-IIP NPs with the precipitation polymerization technique with EGDMA (as the crosslinking agent) and AIBN (as the radical initiator), and then constructed the sensor (IIP/MWCNT-CPE) by thoroughly mixing IIP (7% *w*/*w*), MWCNT (6% *w*/*w*), graphite powder (74.8% *w*/*w*) and paraffin oil (12.2% *w*/*w*) and being transferred to a hole (2.00 mm in diameter, 3 mm in depth) which was located at the end of the electrode body [87]. The synthesis process was simple. Pb^2+^ was adsorbed and reduced to a metal form by square wave anodic stripping voltammetry (SWASV). The results showed that the presence of MWCNTs and IIP nanomaterials in the CPE enhanced the analytical signal of the electrode. The obtained IIP/MWCNT-CPE exhibited a linear range of 1.0 × 10^−11^–8.0 × 10^−8^ M, the detection limit of 3.8 pM (S/N = 3), excellent sensitivity of 20683 A M and high selectivity toward Pb^2+^ over common potential interfering HMIs.

Since the strong van der Waals hinders the dispersion in solution, raw CNTs are rarely used directly to absorb metal ions, and proper surface treatment of CNTs can improve their dispersibility, such as vinyl functionalization CNTs [29].

**Figure 5:**
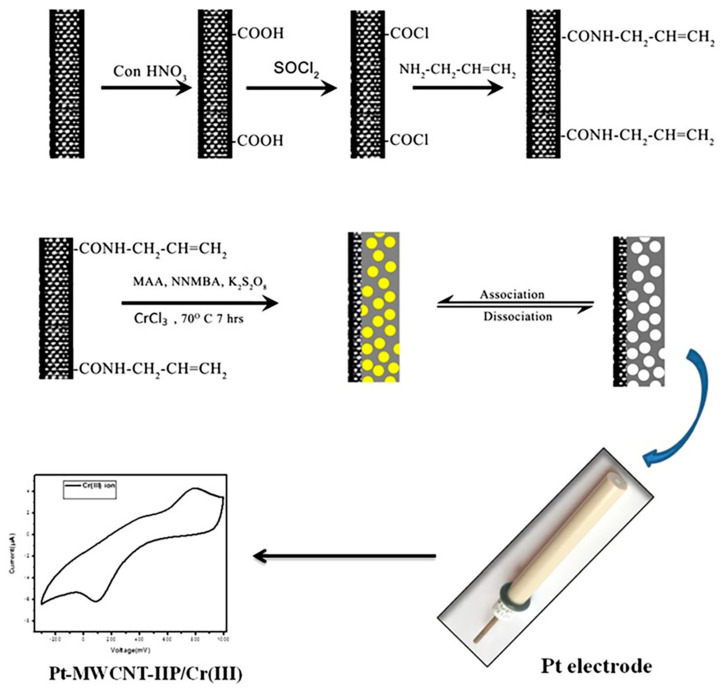
The preparation of Pt-MWCNT-IIP electrode. Reprinted with permission from Ref. [89]. Copyright 2018, John Wiley and Sons.

Mathew et al. designed seven HMIIECS based on vinyl-functionalized MWCNTs. In 2017, they prepared Zn^2+^ IIPs by using AA as monomer and NNMBA as crosslinker layered on vinyl functionalized MWCNTs and the designed electrochemical sensor (MWCNTs-Zn^2+^-IIP) showed great selectivity for Zn^2+^ in the presence of other metal ions. [88]. The electrochemical properties of IIP modified electrodes were studied and optimized by CV. The LOD of Zn^2+^ by DPV was 1.32 × 10^−4^ µM, and this system was suitable for the detection of Zn^2+^ in paint industry wastewater. In 2018, they developed an electrochemical sensor for Cr^3+^ detection based on MWCNT-IIP with high adsorption rate, selectivity and sensitivity [89]. The Cr^3+^ IIP was synthesized by using MAA as the functional monomer, NNMBA as the crosslinking agent, potassium peroxo disulphate as the initiator and Cr (III) ion as the template ion. The sensor reacted quickly to Cr^3+^ ions due to the presence of the MWCNTs nanolayer on IIP with specific binding sites. The preparation process of Pt-MWCNT-IIP was shown in Figure 5. DPV method was used to obtain the LOD of MWCNTs-Cr^3+^-IIP of 0.051 μM. The sensor also showed excellent selectivity, high sensitivity and good reproducibility. At the same time, it was confirmed that the adsorbent had significant selectivity for Cr^3+^ in the presence of other competing metal ions, which had great relevance in the actual sample analysis without interference of other metal ions. The effect of adsorbent on Cr^3+^ in wastewater of metal electroplating industry has been tested effectively. In the same year, they designed a highly selective Co^2+^ electrochemical sensor based on vinyl-functionalized MWCNTs which was also prepared by IIT [90]. The Co^2+^ binding site was formed by coating vinyl-functionalized MWCNTs using Co^2+^ as a template and NNMBA crosslinked polyacrylic acid as solid polymer matrix. The developed system was successfully characterized by different analytical techniques. The selectivity of the system was checked with different metal ions, and the electrochemical response of the nanostructured modified platinum electrode was studied and optimized. The developed MWCNTs-IIP/Co/PE had fast sensing and high selectivity for Co^2+^. The LOD of the sensor was investigated by using DPV and found to be 1.01 × 10^−5^ μM. The practicality of this sensor has been successfully applied for trace sensing and extraction of Co^2+^ from practical samples such as fertilizers and batteries. In another report, they developed an IIECS with good selectivity and high sensitivity for Ni^2+^ using vinyl functionalized MWCNTs, taking into account the advantages of IIT and electrochemical methods [91]. This electrochemical sensor can easily identify Ni^2+^ in the presence of other metal ions, with a LOD of 0.028 µM found by DPV. The practical applicability of the modified electrode was also investigated using real samples collected from lake, steel and electroplating industries. They constructed a Pb^2+^ IIECS (MWCNTs-Pb(II)-IIP) with vinyl-functionalized MWCNTs as the backbone [92]. The characteristics of the developed Pb^2+^ IIECS were investigated by CV and DPV. The study has shown that the developed sensor can detect Pb^2+^ in the presence of other metal ions with a LOD of 2 × 10^−2^ μM. The sensing system was able to successfully identify Pb^2+^ from a variety of actual samples, including lake water, mining wastewater, food samples and environmental samples such as cosmetics, with a recovery rate of 99%. They fabricated another Cd^2+^-templated sensor on vinyl-functionalized MWCNTs [93]. Compared with IIP, MWCNTs-IIP had a higher specific surface area, which was because the presence of MWCNTs can effectively enhance the adsorption of Cd^2+^ on the adsorbent surface. In addition, as for the viewpoint of dispersibility, MWCNTs were easy to aggregate and precipitate at the bottom of the container, while the dispersibility of vinyl-functionalized MWCNTs increased, and the dispersion solution did not change even after 6 months, which was beneficial to increase the specific surface area and enhance the adsorption of Cd^2+^. The calibration curve with LOD of 0.03 μM was obtained by DPV under optimal conditions. Selectivity studies showed that the binding capacity of Zn^2+^, Cu^2+^, and Ni^2+^ was lower than that of Cd^2+^. MWCNTs-IIPs had high selectivity for Cd-Cu mixtures, relatively high selectivity for Cd-Ni mixtures, and low selectivity for Cd-Zn mixtures. In 2019, they synthesized Mn^2+^ IIP and composited with vinyl functionalized-MWCNTs by free radical polymerization to prepare an electrochemical sensor (Pt-MWCNTs-Mn(II)-IIP) for the detection of Mn^2+^ [94]. The LOD of the electrochemical sensor was investigated with DPV and found to be 0.0138 μM, and the imprinted adsorbent showed higher selectivity and specificity for Mn^2+^ compared to other metal ions. The feasibility results of the electrochemical sensor showed that it had a promising application in real lake water samples, pigments, cosmetics and fertilizers. From this, we can summarize one of the advantages of IIT, namely, changing the types of imprinted metal ions based on the same composite materials to detect different HMIs, and to improve the specific recognition of target HMIs detection and reduce LOD.

In 2017, Roushani et al. designed a Mn^2+^-imprinted sensor based on Mn^2+^-IIP particles and MWCNTs-CS-ionic liquid (IL) nanocomposite modified GCE, denoted as Mn(II)-IIP/MWCNTs/CS/IL/GCE [95]. The results showed that the significantly enhanced current signal was observed at Mn(II)-IIP/MWCNTs-CS-IL/GCE, indicating that Mn(II)-IIP/MWCNTs-CS-IL composites had more Mn^2+^ binding sites than other composites (NIP/MWCNTs-CS-IL and MWCNTs-CS-IL). It can be attributed to the synergistic effect of MWCNTs-CS-IL nanocomposites and immobilized Mn(II)-IIP on the GCE surface. The MWCNTs-CS-IL nanocomposites provided enhanced loading sites for the modification of Mn (II)-IIP particles due to their large specific surface area. The conductivity of the sensor increased due to their excellent conductivity. Therefore, the presence of MWCNTs/CS/IL significantly enhanced the electron transfer rate in the redox reaction. The important parameters such as IIP dosage, pre-concentration potential, and enrichment time of the sensor were optimized to 1.0 mg, −1.4 V and 120 s, respectively, with a linear response in the range of 2.0 to 9.0 μM, and the LOD of 0.15 μM.

#### 3.1.2. Graphene Materials

**Figure 6:**
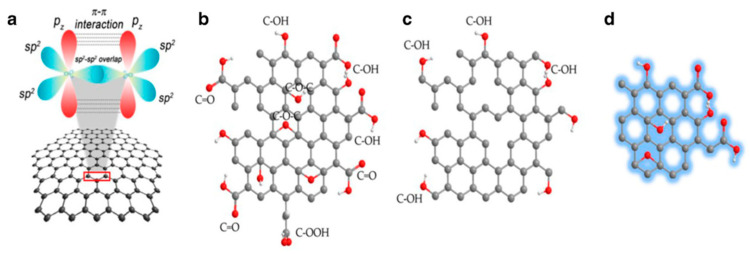
Structure of (**a**) GR, (**b**) GO, (**c**) rGO and (**d**) GQDs. Reprinted with permission from Ref. [99]. Copyright 2021, the Springer Nature.

Graphene (GR) is made up of a single sheet of carbon atoms arranged in sp^2^, which are covalently bonded to each other and arranged in an ordered hexagonal lattice [96]. GR has the characteristics of large specific surface area, high porosity, many active centers, high charge transport rate, good electrical conductivity, high mechanical stability, and good electrocatalytic and photocatalytic activity [97]. GR is prepared by a low-cost, simple, green and environmentally friendly method, which is easy to functionalize compared to metals [98]. Various structures of GR, including graphene oxide (GO), reduced graphene oxide (rGO), GR quantum dots, GR nanoplates, GR nanoribbons, and three-dimensional GRs exhibit various properties and extend the application of GR from energy equipment and water treatment technology to sensors, as shown in Figure 6 [99].

Bai et al. combined the advantages of graphene, IIPs and electrochemical techniques to develop a highly sensitive and selective IIECS (Pd(II)-IIM/GR/GCE) for amperometric determination of Pd^2+^ in complex substrates [100]. In this study, GR was directly modified on the electrode surface by GO electrodeposition, which can enhance the electron transfer and sensitivity of the sensor. Subsequently, IIP was synthesized on this modified surface by in situ polymerization with allylurea (NAU) as a functional monomer, EGDMA as a crosslinking agent, and AIBN as an initiator. By measuring the i-t curve of Pd^2+^, it was found that the linear response range of the designed electrochemical platform to Pd^2+^ was 2.0 × 10^−8^–2.0 × 10^−4^ M, and the LOD was 6.4 × 10^−9^ M. Interference studies have shown that other metal ions did not interfere with the determination of Pd^2+^ even if their concentration was 15 times higher than that of Pd^2+^. The sensor has been successfully used for the determination of Pd^2+^ in the catalysts and factory samples with RSD of less than 3.3% (n = 5) and recoveries in the range of 99.2–106.5%.

In general, the direct use of pristine GR for electrochemical sensing has many problems such as low sensitivity, interference from other substrates, and easy agglomeration. Therefore, GR-based composite nanomaterial sensors have been extensively studied [101].

The sensitivity of HMIs sensing will be increased by using sensor based on polymer modified GR with wealthy reactive sites because in this case the analyte can be preconcentrated on the electrode surface [101]. In 2018, Wu et al. developed an ion-imprinted electrochemical platform (CS-GR-IIP) based on CS-GR nanocomposites for the determination of hexavalent chromium [62]. CS possesses non-toxicity, good biodegradability, superior biocompatibility, favorable flexibility, excellent film forming capacity, and abundant metal chelating sites, so it is very suitable for preparing stable IIM on the electrode surface with various modification materials [102]. The IIPs were prepared by one-step electrodeposition. The electrochemical behavior of CS-GR-IIP was studied by using CV, electrochemical impedance spectroscopy (EIS) and DPV. The linear range of CS-GR-IIP/electrode was 1.0 × 10^−9^–1.0 × 10^−5^ M, and the LOD was 6.4 × 10^−10^ M (S/N = 3). In the presence of Zn^2+^, Co^2+^, Cu^2+^, Ni^2+^, Mn^2+^, MnO_4_^−^, C_2_O_4_^2−^, S_2_O_6_^2−^ and MoO_4_^2−^, the sensor had high selectivity, excellent stability and good repeatability for the detection of Cr^6+^. It has been successfully applied to the detection of Cr^6+^ in tap water and river water.

Noble-metal NPs modified GR has been applied for the construction of HMIIECSs because metal NPs exhibit high catalytic activity through the size effect and GR can transfer the electrons gained during the catalysis of metal NPs to the electrodes, thus speeding up the catalytic process [101]. Among the noble-metal NPs, Au NPs are the most widely used in the detection of metal ions because of their advantages of high chemical stability and easy preparation [101]. Wu et al. developed an ultrasensitive electrochemical platform based on imprinted CS/AuNPs/GR nanocomposites by electropolymerization (electrodeposition for 180 s at −1.2 V) for trace monitoring Cd(II) ions in drinking water and milk samples [25]. The CS/AuNPs/GR/GCE platform showed high sensitivity, excellent selectivity, good stability, and repeatability. Under the optimized conditions, its linear range varied from 0.1 to 0.9 μM and the LOD was 1.62 × 10^−4^ μM. The method was applied for sensing Cd(II) ions in real samples, and the results demonstrated that the developed CS/AuNPs/GR/GCE was a promising electrochemical platform for the quantitative analysis of Cd(II) ions in food samples. Shirzadmehr et al. introduced a novel Zn^2+^-ion selective electrode based on a nano-sensing layer composed of silver NPs decorated GR nanosheets (GNS@Ag NPs), Zn^2+^-IIP NPs, 1-butyl-1-methylpyrrolidine di (trifluoromethyl sulfonyl) imide IL, and graphite powder (GP) [103]. Zn^2+^-IIP NPs as the efficient sensing agent was synthesized by using Zn^2+^ as the template ion, methanol/ethanol (3/1; *v*/*v*) as the porogen solvent, MAA as the monomer, EGDMA as the crosslinker and AIBN as the initiator. Under the optimized experimental conditions, the lower LOD of the proposed potentiometric sensor was 1.93 × 10^−1^ μg/L, and the linear analysis range was from 2.62 × 10^−1^ to 6.54 × 10^−5^ μg/L. The zinc ion-selective sensor proposed in this paper has been successfully applied to the high-sensitivity determination of trace Zn^2+^ in environmental and biological samples.

In the electrochemical analysis of HMIs, GO prepared by the GR precursor system is more common because many oxygen functional groups (such as carboxyl and hydroxyl groups) are introduced into its GR monolayer, which can improve the dispersibility in solutions and provide enough binding sites for adsorbing HMIs [29]. Therefore, many ECSs for HMIs based on GO and its composites have been developed continuously [104].

**Figure 7:**
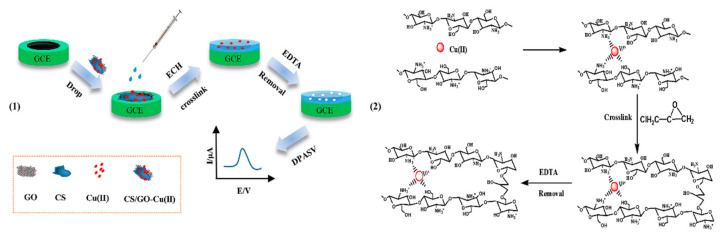
Schematic diagram of the fabrication procedure for CS/GO-IIP/GCE (**1**) and the corresponding chemical processes involved (**2**). Note: the outer green layer is not GCE but the electrode insulation; GCE is the black inner disk. Reprinted with permission from Ref. [65]. Copyright 2019, Elsevier.

In 2018, Yasinzai et al. fabricated Hg(II)-imprinted polymeric- and composite based sensors and investigated their efficiency base on four polymeric systems such as (1) polystyrene, (2) poly(vinylpyrrolidone), and (3) Styrene-co-vinylpyrrolidone polymer, and IIP composite of Styrene-co-vinylpyrrolidone polymer and GR sheets [105]. The ion-imprinted polystyrene system was an ideal receptor for the detection of mercury ions in solution, and the LOD was 2 ppm. The sensitivity of the IIP copolymer system was further improved by recombination with GO, and the LOD of the recombination system was estimated to be about 1 ppm. To sum up, the sensitivity and selectivity of IIPs and composite were in the following order: composite > polystyrene > copolymers > poly(vinyl- pyrrolidone). The results indicated that by combining the IIT with the interdigital transducers, a very sensitive, selective, low-cost and user-friendly sensor system can be made for any HMI. Wei et al. designed a CS/GO-IIP/GCE by the dip coating method for highly sensitive and selective detection of Cu^2+^, and the preparation process was shown in Figure 7 [65]. The Cu^2+^ IIPs were synthesized by dropping CS/GO/Cu^2+^ composite on the GCE surface and chemically crosslinked with epichlorohydrin. The electrical conductivity of the sensor was improved and the electrochemical signal was amplified attributed to the introduction of GO. After optimization, the linear relationship was observed in the range of 0.5–100 μM, and the LOD was 0.15 μM. Conventional metal ions had little interference in the detection of Cu^2+^, and the CS/GO-IIP/GCE showed good reproducibility with an RSD of 3.3% by repeated DPASV. Moreover, the performance of the CS/GO-IIP/GCE was validated in tap and river water samples with acceptable recoveries.

**Figure 8:**
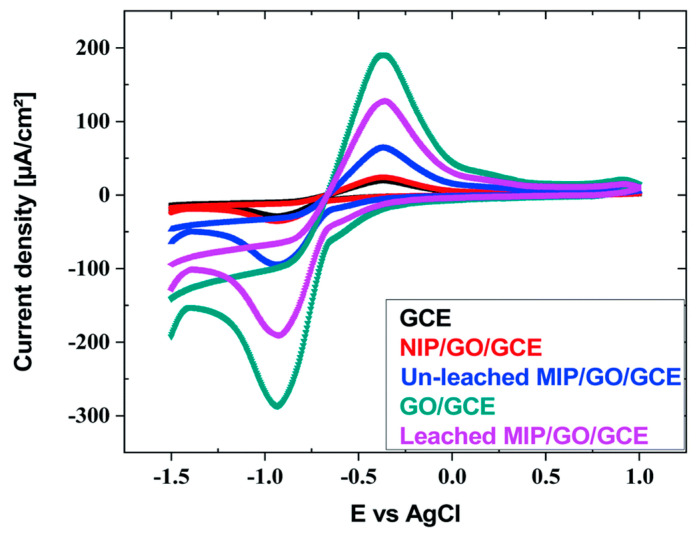
Cyclic voltammograms of GCE, GO@GCE, NIP/GO@GCE, unleached IIP/GO@GCE and leached IIP/GO@GCE in Cd(II) solution buffered with acetate. Reprinted with permission from Ref. [106]. Copyright 2021, the Royal Society of Chemistry.

Abdallah et al. designed a highly selective and sensitive electrochemical sensor based on IIP and GO (IIP/GO@GCE) by a drop coating technique for Cd(II) monitoring in different real samples [106]. The Cd(II)-IIP was successfully prepared using the new complexing agent and functional monomer of ethyl 6-(allyloxy)-2-amino-4-phenyl-4H-benzo[f]chromene-3-carboxylate (EAAP), the initiator of AIBN and the crosslinker of EGDMA. As shown in Figure 8, GO had high conductivity and a large surface area, which facilitated the electron exchange of cadmium ions. Moreover, after fixing the leached IIP, it was found that the peak current intensity increased significantly. The electrochemical response of IIP/GO@GCE for Cd(II) was investigated by ASV, which showed a wide detection range of 4.2 × 10^−12^–5.6 × 10^−3^ M and a very low LOD of 7 × 10^−14^ M. In addition, the new developed sensor also achieved good selectivity, high repeatability and stability.

Topcu et al. developed a Cu^2+^-selective electrode with a highly selective potential response to Cu^2+^ based on GO/MWCNT/Cu^2+^-IIP composite, which was composed of GO (65.0% (*w*/*w*)), MWCNT (5.0% (*w*/*w*)), Cu^2+^-IIP (20.0% (*w*/*w*)), and paraffin oil (10% (*w*/*w*)) [27]. The Cu^2+^-IIP was synthesized by using EGDMA as the crosslinker, AIBN as the free radical initiator, and Cu^2+^-5-methyl-2-thiozylmethacrylamide monomer complex, and was first used as an electroactive component in an ion-selective electrode. The electrode showed a linear response in the concentration range of 1.0 × 10^−6^–1.0 × 10^−1^ M (the correlation coefficient was 0.9998) with the sensitivity of 26.1 ± 0.9 mV decade^−1^ and the LOD of 4.0 × 10^−7^ M. In the range of pH 4.0–8.0, it was stable and reversible with a rapid potential response of 3 s and a lifetime of more than 1 year. Satisfactory results were obtained in potentiometric titration and real water sample determination.

Graphite is oxidized to graphite oxide, subsequently stripped to GO and eventually reduced to rGO. Although both GO and rGO contain oxygen functional groups, such as epoxy, hydroxyl and carboxyl groups, rGO contains less oxygen functional groups after reduction of GO. These oxygen-containing groups influence their electrochemical properties in terms of electron transfer rates or molecular adsorption/desorption, and provide anchor sites for enzymes or other species-specific sensing applications [107]. Although GO and rGO are widely used to detect different target molecules in electrochemistry, rGO shows more advantages in the electrochemical applications. The main characteristic of rGO is that its sp^2^ carbon content is higher than that of GO. The natural result is the presence of a large π orbital, which allows for higher heteroelectron transfer rates than do GO [108].

**Figure 9:**
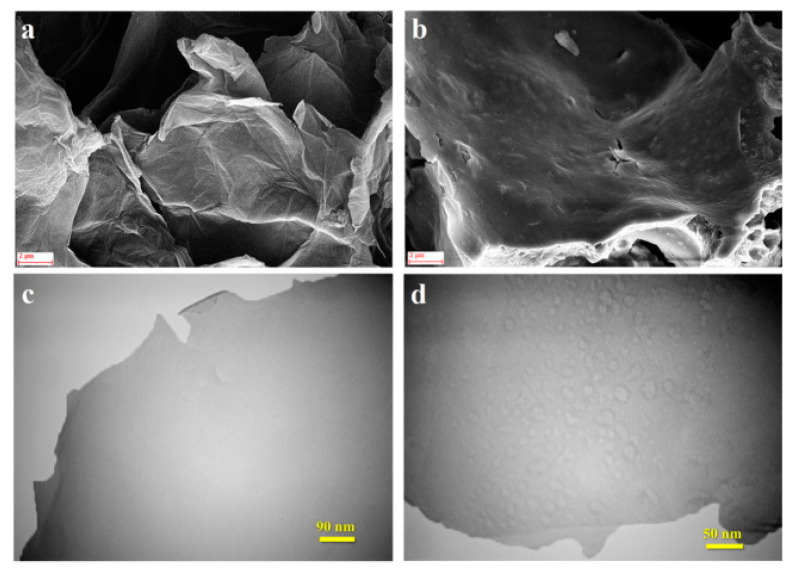
(**a**) FE-SEM image of rGO, (**b**) FE-SEM image of rGO-IIP, (**c**) TEM image of rGO and (**d**) TEM image of rGO-IIP. Reprinted with permission from Ref. [109]. Copyright 2016, Elsevier.

Ghanei-Motlagh et al. prepared IIP on the surface of rGO by surface imprinting technology, using MAA as the functional monomer, EGDMA as the crosslinker, 2,2′-(9E,10E)-1,4-dihydroxy-anthrace-9,10-dimethylene)bis(hydrazi-1-carbonthionamide) (DDBHCT) as the chelating agent and ammonium persulfate (APS) as the initiator [109]. The FE-SEM and transmission electron microscope (TEM) images of rGO and rGO-IIP nanocomposites were shown in Figure 9. rGO-IIP/GCE was used for the detection of Hg^2+^ in a linear range of 0.07–80 μg/L with a LOD of 0.02 μg/L (S/N = 3), lower than the standard of the WHO. The electrochemical sensor had the advantages of high precision, selectivity, precision and reproducibility, and was applicable for the determination of mercury (II) ions in different water samples.

Hu et al. designed two IIECS for both detection of Cd^2+^. One was based on ion-imprinted polypyrrole and reduced graphene oxide (PPy/rGO) composites [67]. The IIP was prepared by a two-step method and used to modify the electrode (IIP/rGO/GCE). Specially, rGO was first electrodeposited on the surface of GCE by CV, and then IIP/rGO/GCE was obtained by electropolymerization of Py and template Cd^2+^ on the surface of rGO, and imprinted template Cd^2+^ were removed by the electrochemical method. In this study, experimental conditions such as deposition time, pH, supporting electrolyte, accumulation time, and potential were optimized. Cd^2+^ was detected by SWASV. The experimental results showed that the sensor had good linearity in the range of 1–100 μg/L, and the LOD was 0.26 μg/L. The sensor exhibited good stability, reproducibility and selectivity, and was triumphantly applied to the determination of trace Cd^2+^ in the practical spiked water samples. Another sensor (PoPD/ERGO/IIP/GCE) was based on Cd^2+^ imprinting, which was prepared by electropolymerization of poly (o-phenylenediamine) (PoPD) and electrochemical reduction of graphene oxide (ERGO) composites, for selective and sensitive determination of trace Cd^2+^ in water [68]. In the range of 1–50 ng/mL, the SWASV dissolution peak showed a good linear relationship with the Cd^2+^ concentration, and the LOD was 0.13 ng/mL (S/N = 3). The PoPD/ERGO/GCE has been successfully applied to the determination of trace Cd^2+^ in real water samples. In contrast, the PPy/rGO/IIP/GCE had a wider linear range, while the PoPD/ERGO/IIP/GCE had a lower LOD. In addition, Shirzadmehr et al. prepared a novel, simple and very sensitive carbon paste sensor by using rGO nanosheets, alumina nanomaterials and Hg^2+^ imprinted polymers as electrode modifiers, and ionic liquids as adhesives [110]. The developed sensor was used for potential determination of Hg^2+^ in a variety of real samples. In order to investigate the ion-electron conversion capability of graphene nanosheets and ionic liquids on the electrode surface, EIS measurements were conducted. After optimization, the LOD of the nanocomposite sensor was 1.95 × 10^−9^ M, and the linear analysis range was 4.00 × 10^−9^–1.30 × 10^−3^ M. Therefore, it was suggested to use the sensor for the determination of Hg^2+^ content in actual samples.

Chen et al. prepared a new IIECS for detecting Cd(II) based on nitrogen-doped rGO (N-rGO)-CS modified GCE via the one step deposition method [33]. The sensor was constructed by co-depositing N-rGO-CS-Cd(II) on the surface of the GCE with Cd(II) as the template ion, N-rGO as the conductivity enhancer, CS as the functional monomer, sodium tripolyphosphate as the crosslinker and ethylene diamine tetraacetic acid (EDTA) as the eluent to remove template ions. Owing to the synergistic effect of N-RGO, the conductivity of the electrode was enhanced, and the detection ability and sensitivity of Cd(II) was also improved ascribed to the target ion holes. The electrochemical properties of Cd(II)-IIECS were evaluated by CV, EIS, and DPV, and the linear range was obtained in 0.01–0.1 μM with the LOD of 3.51 nM (S/N = 3). It also showed that the developed Cd(II)-IIECS had high sensitivity, good reproducibility, well stability and was suitable for practical application.

**Figure 10:**
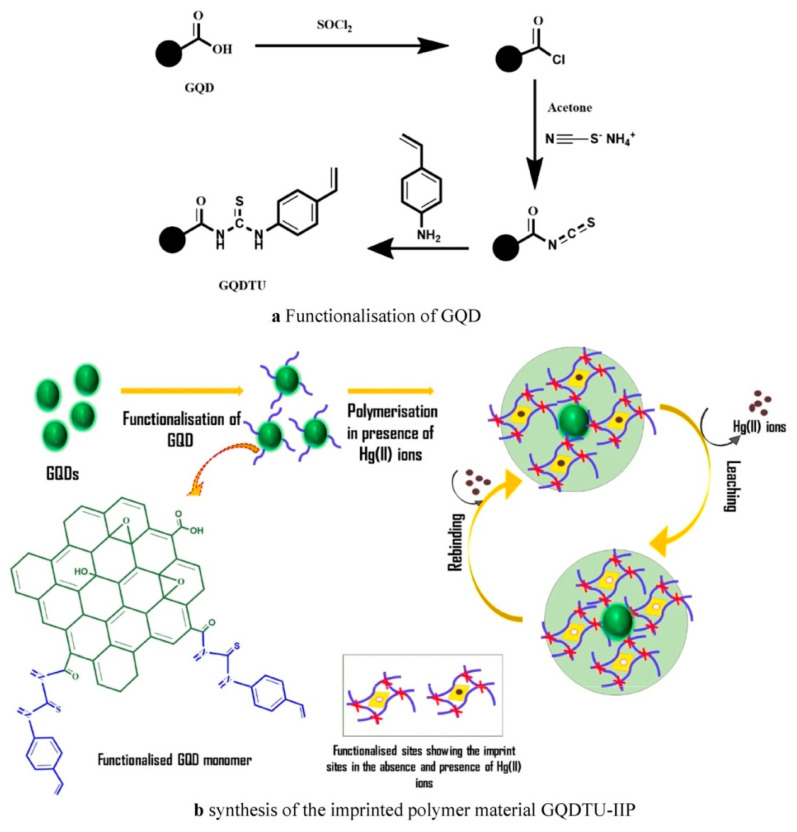
(**a**) Functionalization of GQD (**b**) synthesis of the imprinted polymer material GQDTU-IIP. Reprinted with permission from Ref. [114]. Copyright 2021, the Springer Nature.

Graphene quantum dots (GQDs) consisting of a monolayer or a few layers of graphene are one of the latest frontiers of carbon based nanomaterials due to their excellent and unique properties including low toxicity, high solubility in many solvents, excellent electronic properties, strong chemical inertness, large specific surface area, rich functionalized edge location, good biocompatibility, and low cost and versatility [111,112]. They offer interesting properties for current electrochemical biosensing, as well as their ability to be modified by attractive surface chemistries and other modifiers/nanomaterials [113]. GQDs are highly electroactive and can reduce the overpotential of the analyte; therefore, they can be doped into MIP ECSs to enhance the current response [15]. In 2021, Soman et al. used thiourea derivative-functionalized GQDs (GQDTU) as functional monomers, and prepared mercury ion-imprinted polymer materials (GQDTU-IIP) by using suspension polymerization technology, and with which being used to modify GCE, a novel nanosensor was synthesized for the electrochemical sensing of Hg(II) ions [114]. The preparation process is shown in Figure 10. The introduction of IIPs improved the selectivity of the sensor to Hg(II) ions, and the interference of other HMIS is negligible. CV and DPV were used to detect Hg^2+^. By DPV, the linear response range of Hg^2+^ concentration was 5.0 × 10^−8^–2.3 × 10^−5^ M, and the LOD was 23.5 nM. However, by CV, the linear response ranges of 6 × 10^−8^–8.5 × 10^−7^ M and 1.4 × 10^−6^–7 × 10^−6^ M were obtained, and the LOD was 30.2 nM.

#### 3.1.3. Graphitic Carbonitride Nanomaterials

Graphitic carbonitride (g-C_3_N_4_) is a typical two-dimensional semiconductor with the core composed of carbon and nitrogen atoms of triazine or hydrazine, and has a hexagonal carbon ring layer similar to most carbon materials [115]. It has the characteristics of wide band gap (2.7 eV), easy preparation, good thermal stability, controllable electronic structure, and its 2D layered structure is conducive to charge carrier transfer [116]. The planar conjugated structure also helps to disperse metals or oxides and prevent agglomeration of nanomaterials [117]. Because of its unique physicochemical properties, large surface area, abundant active centers, and nanostructure design, g-C_3_N_4_ is widely used as a catalyst in light-emitting devices (LEDs), photocathodes, and sensors [118].

Reza Ganjali et al. designed an electrochemical sensor for Hg^2+^ by composing IIP and g-C_3_N_4_ [119]. Hg^2+^-IIP nanomaterials were prepared by precipitation polymerization using a functional monomer (itaconic acid, ITA), then was used to modify the CPE together with g-C_3_N_4_, and the modified electrode was employed for Hg^2+^ analysis. The results obtained by SWASV method were compared with those obtained by unmodified CPE, and it was found that modified CPE had a higher propensity for Hg^2+^. The modified electrode was found to have a suitable linear response in the range of 0.06 to 25.0 nM, with an excellent LOD of 18 pM (S/N = 3). A detailed list of carbon nanomaterials-based HMIIECSs for various HMIs was given in Table 3.

### 3.2. Metal Nanomaterials

When the metal-based nanomaterials used for imprinting are reduced to nanometer size, the metal exhibits many unique properties that the bulk metal atoms do not have, among which gold nanomaterials are the most explored [30].

#### Gold Nanomaterials

Gold nanomaterials (AuNPs) have quasi-spherical polycrystalline gold nanostructures with diameters ranging from 5 to 100 nm, which have excellent properties and unique advantages (such as high catalytic activity, good biocompatibility and fast electron transfer speed, high stability, facile synthesis method, naked eye visibility, shape- and size-dependent optical properties, and direct surface modification) [120,121]. More interestingly, the inherent properties of AuNPs can be controlled by tweaking their shape, size and chemical environment. In addition, AuNPs of different shapes, such as spheres, rods, triangles, cubes and nanowires, have been prepared by various synthesis techniques [122].

**Figure 11:**
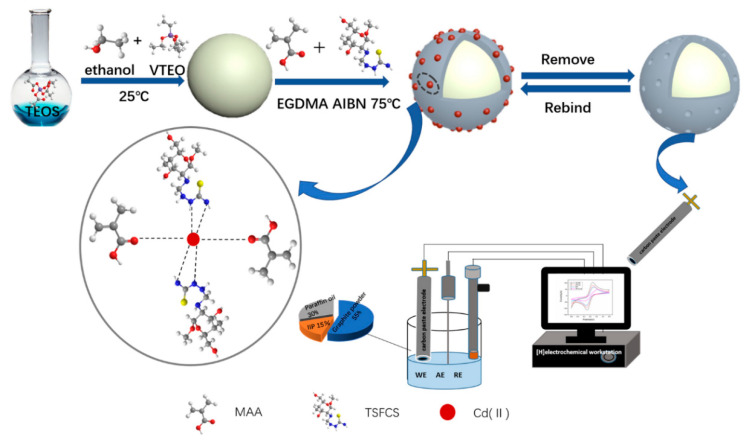
Schematic diagram of the preparation process of Cd^2+^ imprinted polymers. Reprinted with permission from Ref. [123]. Copyright 2021, the Springer Nature.

**Figure 12:**
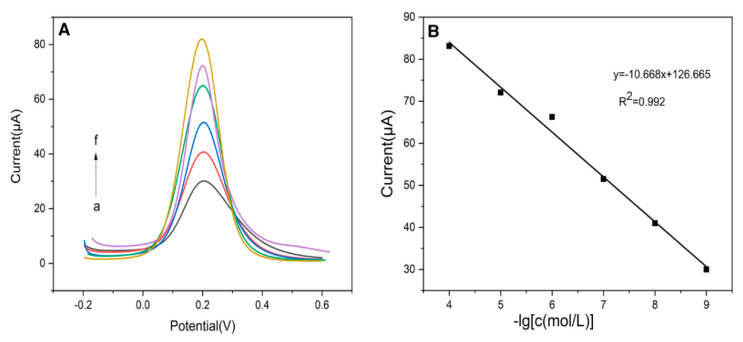
(**A**) DPV of IIP electrode after elution in different concentration of Cd^2+^ and (**B**) calibration curve. Reprinted with permission from Ref. [123]. Copyright 2021, the Springer Nature.

Huang et al. prepared a new electrochemical sensor (see Figure 11 for the preparation process) based on IIP and AuNPs for selective and sensitive determination of Cd^2+^ in the actual samples [123]. In this study, the IIP was surface imprinted on the silicon sphere to increase the quantity of the imprinting site and enhance the adsorption performance of the imprinted material by selecting thiosemicarbamide functionalized CS and MAA as functional monomers, and doped with AuNPs, which widened the electrochemically active site and accelerated the transfer of electrons from the reaction center to the electrode surface. The electrochemical behaviors of the IIP composites were characterized by CV, DPV and EIS, and the results showed that the as-prepared sensor exhibited good selectivity to the target Cd^2+^. DPV was used to discuss the relationship between the peak current and Cd^2+^ concentration under optimal conditions. As shown in Figure 12, the current increased linearly with Cd^2+^ logarithmic concentration from 10^−9^ to 10^−4^ M. The correlation coefficient was 0.992, and the LOD was 1.43 × 10^−10^ M. The improved sensor not only had good selectivity and high sensitivity, but also had good repeatability and stability.

Wu et al. developed a reliable and disposable electrochemical sensor for sensitive and selective detection of lead ions, which was denoted as imprinted CS/AuNPs/GR/Nafion/MWCNTs/GCE (Pb^2+^-IIP sensor) [66]. The sensing platform was constructed by firstly immobilizing MWCNTs on the surface of GCE, drop coated Nafion, subsequently deposited CS incorporating AuNPs and GR through electrodeposition combining ion imprinting. The Pb^2+^-IIP sensor displayed an advanced performance owing to the synergistic effects from hierarchically nanostructure, including the inner layer of Nafion/MWCNTs which enhanced the electronic conductivity and facilitated the charge transfer to the electrode, the imprinted CS/AuNPs/GR outer layer which provided more recognition sites and pathways for Pb^2+^, and the NPs which greatly improved the electroactive surface and layed nano-micro porous structure. The proposed sensor exhibited sensitive determination of Pb^2+^ with high repeatability, good reproducibility and stability. It showed a linear range of 1.0 × 10^−9^–5.0 × 10^−5^ M with a LOD of 2.83 × 10^−10^ M under optimized conditions, and has been successfully applied to the determination of Pb^2+^ in water and milk samples.

Nanoporous gold (NPG) has attracted many researchers because of its large specific surface area, high conductivity, good catalytic activity and biocompatibility [124]. NPG can be combined with MIP to construct a simple electrochemical sensor, which has great potential to improve the sensitivity of electrochemical sensor by combining their advantages [125].

Ma et al. developed an electrochemical sensor based on IIP and NPG modified gold electrode (IIP/NPG/GE) for the determination of As^3+^ in different types of water [71]. NPG had the characteristics of high conductivity, large specific surface area, and high biocompatibility and was electrochemically deposited on the electrode surface of GE by the Tominaka method. The layer of IIP was obtained on the surface of NPG/GE by employing As^3+^ as the template and OPD as the functional monomer via in situ electropolymerization. CV and EIS were utilized to study the electrochemical performance of IIP/NPG/GE, which had good stability and selectivity, and showed a linear range for As^3+^ of 2.0 × 10^−11^–9.0 × 10^−9^ M and the LOD of 7.1 × 10^−12^ M (N = 3). It has been successfully applied to the determination of As^3+^ in four kinds of water.

### 3.3. Metal Oxide Nanomaterials

Metal oxide nanomaterials are suitable for analytical applications due to their excellent properties, including high selectivity and sensitivity, and are considered to be one of the most important materials in high-performance electronics and environmental monitoring [126]. Zinc oxide (ZnO) nanostructures have attracted much attention due to their attractive photoelectric properties and great structural diversity, and they have a wide range of applications in various industrial fields, such as field effect transistors, lasers, photodetectors, solar cells and batteries, chemical and biological sensors [127]. Ait-Touchente et al. developed an electrochemical platform for Hg^2+^ detection based on Au/ZnO/IIP/GE [70]. The biomimetic IIP was prepared by electropolymerizing Py in the presence of L-cysteine as a chelating agent and Hg^2+^ (template) on the GE surface modified with vertically grown ZnO nanorods. The strategy of combining diazonium salt modification and ZnO nanorod decoration of GE increased the specific surface area considerably and thus the sensor performance was improved. The LOD of the designed sensor was ~1 pM, and Au/ZnO/IIP/GE was highly selective for Hg^2+^ compared with Cd^2+^, Pb^2+^ and Cu^2+^. This sensor design can open new horizons for monitoring toxic HMIs in water, thereby helping to improve environmental quality.

### 3.4. Magnetic Ferric Oxide Nanomaterials

Magnetic nanomaterials (MNMs) are considered promising support materials in analytical chemistry and have been used in separation/extraction and sensor/biosensor applications. Their wide application is mainly due to their superparamagnetism, low toxicity, high biocompatibility and high surface volume ratio [128]. Magnetic material is mainly used as a kind of nanomaterial to improve the morphology and sensitivity of MIP based sensor interface. In the construction of sensing interfaces, after the modification of magnetic materials or magnetic composites, MIP films are usually constructed by electropolymerization due to its simple and effective operation. Sol-gel electrodeposition is also a potential method to prepare MIP films and improve MIP properties, which has been used to construct MIP films at the interface of magnetic molecularly imprinted electrochemical sensors (MMIECS) [129].

As an ideal adsorbent, Fe_3_O_4_ nanomaterials have been widely used as ECSs for the detection of HMIs, and the quasi-spherical Fe_3_O_4_ nanomaterials with an average diameter of about 10 nm can be used for the independent detection of multiple HMIs [130]. Ghanei-Motlagh and Taher prepared a voltammetric sensor (Fe_3_O_4_@SiO_2_@IIP@CPE) for selective detection of Ag^+^ base on magnetic Ag^+^-IIP NPs, which was achieved by imprinting Ag^+^ on silica-coated magnetic NPs (Fe_3_O_4_@SiO_2_) in the presence of PAN, MAA, EGDMA, APS and AgNO_3_ [131]. DPV was employed to measure pre-enriched Ag^+^ on the surface of Fe_3_O_4_@SiO_2_@IIP@CPE. Under optimal conditions, it showed a linear response in the concentration range of 0.05–150 μg/L with a LOD of 15 ng/L, remarkable selectivity and good reproducibility (RSD of 4.7%).

Ghanei-Motlagh et al. prepared IIP NPs on vinyl functionalized magnetic Fe_3_O_4_ NPs with the surface imprinting process and combined it with MWCNTs to modify CPE for monitoring Pb^2+^ [132]. The best response to Pb^2+^ (the concentration range of 3–55 μg/L with a low LOD of 0.5 μg/L) was obtained when the CPE was composed of 53% (*w*/*w*) of graphite powder, 10% of MWCNTs, 7% (*w*/*w*) of Pb^2+^-IIP, and 30% (*w*/*w*) of paraffin oil. The designed electrochemical sensor also displayed remarkable selectivity and good reproducibility with RSD of 3.1%. It was applied to determine the content of Pb^2+^ in different environmental water samples, and the results were confirmed by graphite furnace atomic absorption spectrometry (GF-AAS).

**Figure 13:**
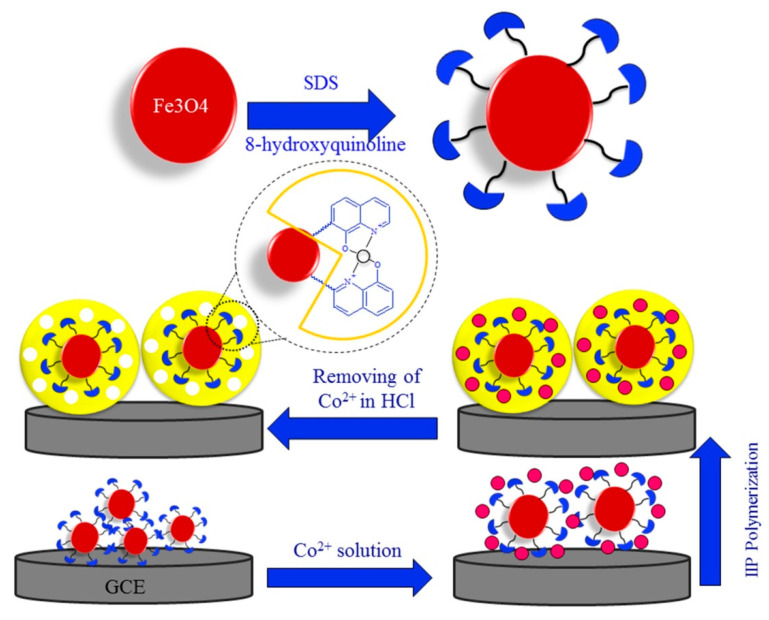
The modification process of Co(II)-IIP-Fe_3_O_4_ electrode. Reprinted with permission from Ref. [61]. Copyright 2017, Elsevier.

Torkashvand et al. developed a novel electrochemical sensor for determination of Co^2+^ based on IIT directly onto the surface of functionalized magnetic NPs (MNPs) modified GCE [61]. The IIP nanobeads was polymerized with AAM as the functional monomer, NNMBA as the crosslinker and APS as the initiator, and around the complex between Co^2+^ and 8-hydroxyquniline groups linked to the casted MNPs at the surface of GCE. Grafting IIP onto the functionalized MNPs improved the specific surface area and adsorption capacity. Preparation of the sensor is shown in Figure 13. Under optimal conditions, the sensor showed a linear range of 0.5 to 20 nM and 20 to 500 nM with a LOD of 0.1 nM. The sensor had the advantages of simple design, short measurement time, high accuracy, a large amount of analysis, low LOD, and good selectivity. It has been successfully applied to the determination of low level of Co^2+^ in different practical samples.

In 2018, Dahaghin et al. developed a new type of nanocomposite (GO@Fe_3_O_4_@benzothiazole-2-carboxaldehyde) to modify GCE—using magnetic graphene oxide (GO@Fe_3_O_4_) and introducing benzothiazole-2-carbaldehyde (2-CBT) for the first time, and simultaneously detecting trace Pb^2+^ and Cd^2+^ in aqueous solution by SWASV [133]. Under the best conditions, the LOD of Pb^2+^ and Cd^2+^ were 0.03 ng/mL and 0.02 ng/mL, respectively. This sensor was a kind of high selectivity and sensitivity sensor, which can be used for the determination of Pb^2+^ and Cd^2+^ in different water samples. In 2020, they grafted a novel IIP on Fe_3_O_4_ NPs, namely 2-(2-aminophenyl)-1H benzimidazole as a novel ligand for the synthesis of IIP for the establishment of Fe_3_O_4_-IIP-GCE for determination of Pb^2+^ content in complex samples [134]. The constructed electrochemical platform exhibited linearity over a wide linear concentration range (0.1–80 ng/mL), low LOD (0.05 ng/mL), high selectivity, convenient use, and low cost. With the advantages of fast response speed and high sensitivity, it can be used for the determination of Pb^2+^ in natural water and fruit juice.

An et al. prepared the Fe_3_O_4_/C/Cu(II)/IIPs/GCE for efficient detection of Cu^2+^ [135]. First, the surface of carbon spheres was modified with Fe_3_O_4_, and then carbon-based ion-imprinted materials were synthesized by IIT. The carbon imprinted material can improve the conductivity, and the magnetic properties of the Fe_3_O_4_ matrix enabled the material to be selectively identified and quickly restored. Therefore, Fe_3_O_4_/C/Cu(II)/IIPs/GCE can detect Cu^2+^ rapidly and selectively. In this study, the electrochemical performance of Cu(II)/IIPs/GCE was evaluated by DPV. The LOD was 5.99 × 10^−6^ M (S/N = 3), which can meet the detection requirements of Cu^2+^ in water. This method provided a new idea for the detection of metal ions in water. It can also be used for rapid selective removal and recovery of Cu^2+^ in water.

### 3.5. Silica Nanomaterials

Silica is the most commonly used core material for the preparation of MIPs because of its advantages such as thermal stability, biocompatibility, high permeability, and stability under acidic conditions, and its surface modification can be achieved by several silane coupling agents such as 3-aminopropyltriethoxysilane (APTES), methacryloxypropyltrimethoxysilane (MPS), and vinyltriethoxysilane (VTES) via conjugation the active hydroxyl groups on the silica particle surface [136]. These modification groups can improve the reactivity of silica surface, which is beneficial to receive IIP shell.

Khairnar et al. prepared Zn(II) IIP by radical polymerization on the surface of vinyl silica particles and applied them to fabricate CPEs for monitoring Zn^2+^ by DPV [137]. Vinyl functionalized silica particles were synthesized using the sol-gel method and the IIP particles were prepared by radical polymerization. Different morphological and elemental techniques were employed for their characterization. The ion-imprinted particles were used to fabricate CPEs as Zn^2+^ sensors. The improved Zn^2+^ sensor showed a linear response in the concentration range of 6.12 × 10^−9^–4.59 × 10^−8^ M, and the LOD and LOQ of the electrode were 1.351 × 10^−8^ M and 4.094 × 10^−8^ M, respectively. The proposed sensor had high selectivity and sensitivity to trace Zn^2+^, and can be used for the detection of Zn^2+^ in aqueous solution.

**Figure 14:**
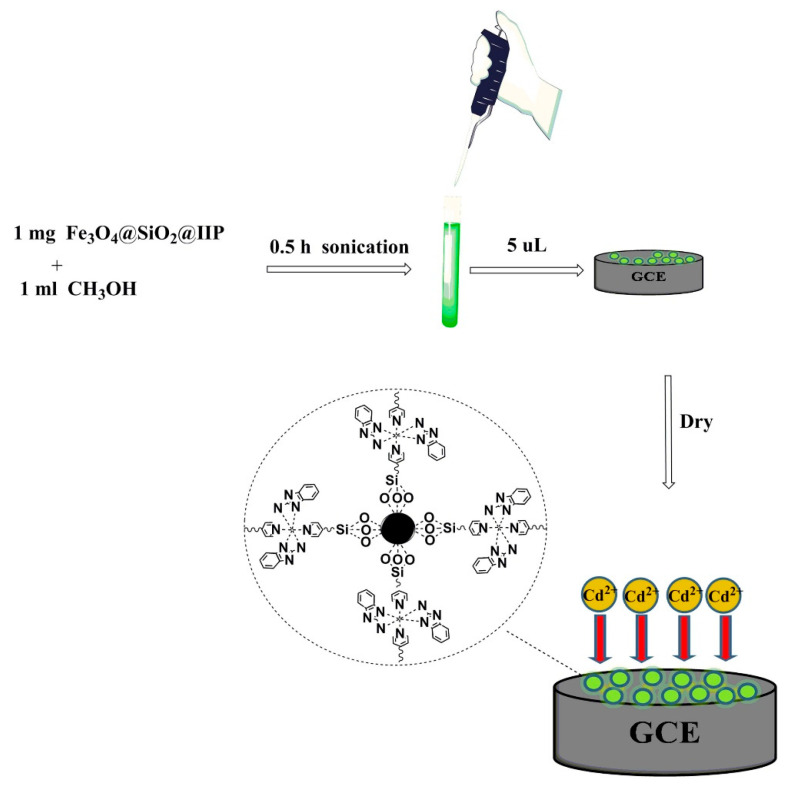
Schematic diagram of Fe_3_O_4_@SiO_2_@IIP modified GCE. Reprinted with permission from Ref. [138]. Copyright 2018, Elsevier.

Dahaghin et al. proposed an electrochemical sensor based on Fe_3_O_4_@SiO_2_@IIP modified GCE for the determination of Cd^2+^ by DPV [138]. Fe_3_O_4_@SiO_2_@IIP was prepared by using the copolymerization approach including cadmium ions, EGDMA, AIBN, 4-VP, and 2-aminobenzimidazole. Fe_3_O_4_@SiO_2_@IIP modified GCE as shown in Figure 14. By grafting IIP onto the functional MNPs, the advantages of combining IIP with core-shell Fe_3_O_4_@ SiO_2_ NPs as well as their relative surface area and adsorption capacity were improved. The linear range of response to Cd^2+^ was 0.008–0.05 μM, and the LOD was 1 × 10^−4^ μM. The platform has been successfully applied to the determination of trace Cd^2+^ in environmental water samples.

Wei et al. homogeneously coated SiO_2_ on the Fe_3_O_4_ core to form the Fe_3_O_4_/SiO_2_ NPs through a revised Stöber method, then crosslinked the CS film incorporating Cu^2+^ to Fe_3_O_4_/SiO_2_ with glutaraldehyde to obtain Fe_3_O_4_/SiO_2_/CS/IIP, and finally dispersed the above Cu^2+^-IIP nanocomposites in Nafion solution and modified GCE by dip-coating to fabricate the electrochemical sensor denoted as Fe_3_O_4_/SiO_2_/CS/Nafion/GCE IIP [47]. During the electrochemical measurement, different experimental parameters were optimized. The sensor showed a wide linear detection range of 0.01–20 μM, a low LOD of 5 nM, and a significant reproducibility with RSD of 3.3%. The research showed that the sensor based on Fe_3_O_4_/SiO_2_/CS imprinted polymer had the potential to monitor the pollution of other toxic metal ions in a harsh environment.

Afkhami et al. synthesized a new nanometer IIP with ion recognition ability using SiO_2_-coated magnetite nanomaterials (SCMNPs) as the core and supporting material of the nanostructure and it was applied to modify CPE for Hg^2+^ detection [139]. Highly selective and sensitive interactions between nano-IIP and Hg^2+^ ions enhanced the deposition of target ions on the electrode surface, thereby enhancing the electrochemical signal. Square wave stripping voltammetry (SWV) was used for the rapid, simple, accurate, sensitive and highly selective determination of Hg^2+^. The linear range and LOD were 0.20–1600.0 ng/mL, 0.04 ng/mL, respectively. The influence of different cations and anions on the determination of target ions was studied, and the electrode was found to be highly selective for the determination of Hg^2+^. In addition, this method has been successfully applied to the determination of Hg^2+^ in water and some food samples.

Wang et al. developed an all-solid-state polyvinylchloride (PVC) membrane cesium potentiometric microsensor based on Cs(I)-MIIP, which was synthesized by the surface-imprinting technique with functionalized magnetic Fe_3_O_4_@SiO_2_ microspheres as the supporter, Cs(I) ion as the template and carboxymethyl CS as the functional monomers [140]. The Cs(I)-MIIP was used as an ion carrier in the construction of the PVC membrane electrode to obtain a selective potentiometric microsensor for Cs^+^. This was the first time to report the electrochemical determination of Cs^+^ by IIP. The prepared sensor showed a highly sensitive response in the concentration range of 1μM to 0.1M. The response time was quite short (less than 3 s), and the LOD was determined to be 0.3μM. The microsensor worked effectively in the pH range of 4–6 and showed ideal selectivity for many other cations including alkali, alkaline earth and heavy metals. It can also be used for the determination of Cs^+^ content in the environmental water samples. The potentiometric results were consistent with those obtained with the ICP-MS method.

Afkhami proposed a new chemically modified CPE (IIP@SiO_2_@Fe_3_O_4_) which can be used for the accurate, simple, sensitive and selective determination of Mo^6+^ [141]. IIP@ SiO_2_@Fe_3_O_4_ was prepared by the sol-gel process. The proposed sensor obtained high sensitivity and a wide linear range for determining Mo^6+^ attributed to sensitive and selective interaction between the target ion and nano-IIP at the surface of the IIP@SiO_2_@Fe_3_O_4_ electrode. The results showed that the linear dynamic range and the LOD were 0.2–500.0 ng/mL and 0.04 ng/mL, respectively. The response reproducibility and stability of the modified electrode were investigated. By investigating the influence of different cations and anions on the determination of target ions, it was found that the electrode had a high selectivity for the determination of Mo^6+^. This method was also used for the determination of Mo^6+^ in actual samples.

The nanomaterials-based HMIIECSs for the detection of Pb^2+^, Cd^2+^, Cu^2+^ and Hg^2+^ in real samples were overviewed in Table 4.

## 4. Conclusions and Future Outlook

**Figure 15:**
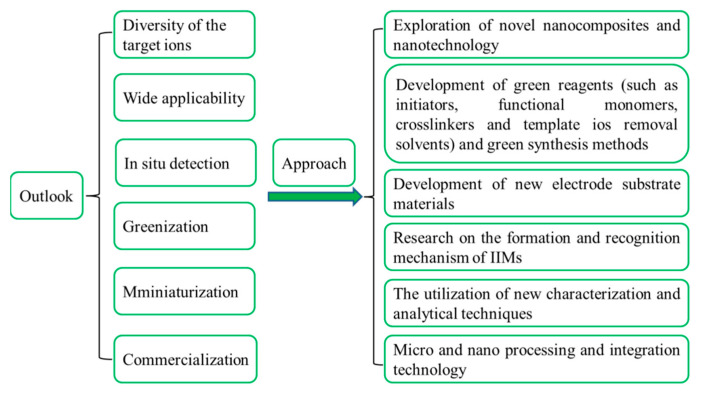
The future outlook and development trends of HMIIECSs.

Rapid and efficient quantitative detection of trace HMIs is an important issue for health and environmental safety. The combination of IIT and electrochemical sensing gives IIECSs remarkable properties (such as high sensitivity, excellent selectivity, strong thermal/mechanical stability, low LOD, facile preparation, and reusability, etc.), which makes them excellent candidates for efficient tools for on-site monitoring HMIs, meeting the growing demand. Nonetheless, IIECSs still need to overcome the shortcomings of IIPs due to their small binding capacity, slow mass transfer rate and low recognition sites. Nanomaterials have unique characteristics such as surface functionalization convenience, high electrochemical activity, good compatibility and high electron transfer performance. The integration of nanomaterials with IIECSs can improve the sensitivity, selectivity, stability, LOD and field detection ability of the sensor for detecting HMIs. This review introduced the preparation and sensing mechanism of the nanomaterials-based HMIIECSs, as well as the recent applications of the nanomaterials in this kind of sensors. The nanomaterials-based HMIIECSs exhibit the characteristics of wide concentration range, low LOD, good selectivity, fast response and accurate results in the detection of HMIs, and have become a new focus of research. Many application examples have demonstrated the great potential of IIECSs in the quantitative detection of HMIs with high sensitivity and high selectivity in many real samples. However, there are still some deficiencies and challenges that need to be addressed, such as few electrode types, limited reuse times, limitations of the target testing HMIs, inadequate field applicability, etc. Important efforts are still required to make in the following aspects: (1) More functional monomers and multifunctional monomers need to be developed to improve the sensitivity of HMIIECSs; (2) New nanocomposites are emerging at an increasing speed and will be used in HMIIECSs; (3) Efforts will be made to develop HMIIECSs for simultaneous analysis of multiple HMIs; (4) Robust HMIIECSs for sensitive and selective detection of HMIs via green approaches are worthy of research and application; (5) It is particularly important to further study the formation and recognition mechanism of IIMs with the help of new technology and characterization techniques, especially the HMIs detection mechanism applied in complex water environments and biological samples; (6) The reusability of HMIIECSs and their applicability in complex environments should be explored to facilitate commercial applications; (7) New types of electrodes, such as wide and flat substrate materials (cellulosic paper, electric glass sheets, etc.) need to be developed and modified with IIPs and NPs to achieve better selectivity and electrocatalytic capabilities, thereby improving the commercialization and convenience of use of HMIIECSs; and (8) Exploring new materials and fabrication methods to obtain fast, micro and integrated portable HMIIECSs suitable for field applications will remain a challenging issue in the coming years. Figure 15 summarizes the future outlook and development trends of HMIIECSs.

## Figures and Tables

**Table 1:** Summary of the HMIIECSs prepared by in-situ polymerization.

Target Ion	Electrode	Nanomaterial	Monomer	Electrochemical Method	Linear Range (μM)	LOD (μM)	Ref.
Co^2+^	GCE	Fe_3_O_4_	AAM	DPCSV	5.0 × 10^−4^–0.02 and 0.02–0.5	1.0 × 10^−4^	[61]
Cr^6+^	GE	GR	CS	DPV	1.0 × 10^−3^–10	6.4 × 10^−4^	[62]
Ce^4+^	SPCE ^a^	Fe_3_O_4_	But-2-enedioic acid bis-[(2-amino-ethyl)-amide]	DPASV	1.78 × 10^−3^–4.45 × 10^−2^	5.0 × 10^−4^	[63]
Gd^3+^	SPCE	Fe_3_O_4_	But-2-enedioic acid bis-[(2-amino-ethyl)-amide]	DPASV	4.71 × 10^−3^–6.02 × 10^−2^	1.2 × 10^−4^	[63]
Zn^2+^	PGE ^b^	MWCNTs	BAAP ^c^	DPASV	4.21 × 10^−4^	1.5 × 10^−3^–0.364	[64]
Cu^2+^	PGE	MWCNTs	BAAP	DPASV	2.5 × 10^−4^	1.54 × 10^−3^–0.375	[64]
	GCE	GO	CS	DPASV	0.5–100	0.15	[65]

SPCE ^a^: Screen-printed carbon electrode; PGE ^b^: Pencil graphite electrode; BAAP ^c^: Bis-(2-acryloylamino-ethyl)-phosphinic acid.

**Table 2:** Summary of the HMIIECSs prepared by electropolymerization.

Target Ion	Electrode	Nanomaterial	Monomer	Electrochemical Method	Linear Range (μM)	LOD (μM)	Ref.
Pb^2+^	GCE	AuNPs/GR/MWCNTs	CS	DPV	1.00 × 10^−3^–50	2.83 × 10^−4^	[66]
Cd^2+^	GCE	AuNPs/GR	CS	DPV	0.10–0.90	1.62 × 10^−4^	[25]
	GCE	RGO	PPy	SWASV	8.90 × 10^−3^–0.89	2.30 × 10^−3^	[67]
	GCE	ERGO	PoPD	SWASV	8.90 × 10^−3^–0.44	1.20 × 10^−3^	[68]
	GCE	N-rGO	CS	DPV	0.01–0.10	3.51 × 10^−3^	[33]
Hg^2+^	GCE	AuNPs/SWCNTs	Mercaptobenzotriazole	DPASV	4.00 × 10^−4^–9.60 × 10^−2^	8.00 × 10^−5^	[69]
	GE	Au/ZnO	Py	SWV	——	~1 × 10^−6^	[70]
As^3+^	GE	NPG	P-phenylened-iamine	CV	2.00 × 10^−5^–9.00 × 10^−3^	7.10 × 10^−6^	[71]

**Table 3:** Carbon nanomaterials-based HMIIECSs for the detection of HMIs.

EC Sensing Platform	Technique	Metal Selectivity	Detection Limit	Linear Range	Ref.
GCE–IIP–MWCNTs	DPV	Hg^2+^	5.0 nM	1 × 10^−8^–7.0 × 10^−4^M	[81]
Tl-IP-MWCNT-CPE	DPASV	Tl^+^	0.76 ng/mL	3.0–240 ng/mL	[82]
CPE-IIP-MWCNTs	——	Cr^3+^	5.9 × 10^−7^ M	1.0 μM–1.0 M	[84]
Ce-IIP-MWCNTs	SWV	Ce^3+^	10.0 pM	1.0 μM–25 pM	[83]
Ag-IIP- MWCNTs	DPV	Ag^+^	1.2 × 10^−10^ M	0.5 × 10^−9^–2.8 × 10^−7^ M	[85]
GCE–IIP–MWCNTs	DPV	Pb^2+^	0.16 μg/L	5.0–10.0 μg/L	[86]
IIP–MWCNTs-CPE	SWASV	Pb^2+^	3.8 pM	1.0 × 10^−11^–8.0 × 10^−8^ M	[87]
MWCNT-CH=CH_2_-IIP	DPV	Zn^2+^	1.32 × 10^−4^ µM	1–5 ppm	[88]
MWCNT-CH=CH_2_-IIP	DPV	Cr^3+^	0.051 μM	1–5 ppm	[89]
MWCNT-CH=CH_2_-IIP/PE	DPV	Co^2+^	1.01 × 10^−5^ μM	1–5 ppm	[90]
MWCNT-CH=CH_2_-IIP/Pt	DPV	Ni^2+^	0.028 µM	1–5 ppm	[91]
MWCNT-CH=CH_2_-IIP	DPV	Pb^2+^	2 × 10^−2^ μM	1–5 ppm	[92]
MWCNT-CH=CH_2_-IIP/Pt	DPV	Cd^2+^	0.03 μM	1–5 ppm	[93]
MWCNT-CH=CH_2_-IIP/Pt	DPV	Mn^2+^	0.0138 μM	1–5 ppm	[94]
IIP/MWCNT/CS/IL/GCE	SWASVs	Mn^2+^	0.15 μM	2.0–9.0 μM	[95]
GR-IIM-GCE	i-t	Pd^2+^	6.4 × 10^−9^ M	2.0 × 10^−8^–2.0 × 10^−4^ M	[100]
CS-GR-IIP	DPV	Cr^6+^	6.4 × 10^−10^ M	1.0 × 10^−9^–1.0 × 10^−5^ M	[62]
CS/AuNPs/GR/GCE	DPV	Cd^2+^	1.62 × 10^−4^ μM	0.1–0.9 μM	[25]
Ag-GR-IIP-CPE	EMF	Zn^2+^	1.93 × 10^−1^ μg/L	2.62 × 10^−1^–6.54 × 10^−5^ μg/L	[103]
GO-IIP-IDE	——	Hg^2+^	1 ppm	——	[105]
CS/GO-IIP	DPASV	Cu^2+^	0.15 μM	0.5–100 μM	[65]
GO–IIP-GCE	ASV	Cd^2+^	7 × 10^−14^ M	4.2 × 10^−12^–5.6 × 10^−3^ M	[106]
GO/MWCNT/IIP	——	Cu^2+^	4.0 × 10^−7^ M	1.0 × 10^−6^–1.0 × 10^−1^ M	[27]
RGO–IIP	SWASV	Hg^2+^	0.02 μg/L	0.07–80 μg/L	[109]
PPy/rGO/IIP	SWASV	Cd^2+^	0.26 μg/L	1–100 μg/L	[67]
PoPD/ERGO/IIP/GCE	SWASV	Cd^2+^	0.13 ng/mL	1–50 ng/mL	[68]
Al_2_O_3_/rGO/IIP	——	Hg^2+^	1.95 × 10^−9^ M	4.00 × 10^−9^–1.30 × 10^−3^ M	[110]
N-rGO-CS-IIP	DPV	Cd^2+^	3.51 nM	0.01–0.1 μM	[33]
GQDTU-IIP	DPV	Hg^2+^	23.5 nM	5 × 10^−8^M–2.3 × 10^−5^ M	[114]
GQDTU-IIP	CV	Hg^2+^	30.2 nM	6 × 10^−8^M–8.5 × 10^−7^ M and 1.4 × 10^−6^ M–7 × 10^−6^ M	[114]
g-C_3_N_4_/IIP	SWASV	Hg^2+^	18 pM	0.06–25.0 nM	[119]

**Table 4:** Overview on nanomaterials-based HMIIECSs for the detection of several common HMIs.

Target Ion	Electrochemical Sensor	Monomer	Crosslinker	Initiator	Imprinting Method	Detection Technique	Linear Range	LOD	Sample	Maximum Permissibe Limit	Ref.
Pb^2+^	MWCNTs/GCE	MAA	EGDMA	AIBN	Precipitation polymerization	DPV	5.0–10.0 μg/L	0.16 μg/L	Drinking water, physiologi-cal serum (NaCl 0.9% *m*/*v*),and synthetic urine	10.0 μg/L (Drinki-ng water), 0.03 mg/Kg (Fruit juice)	[86]
	MWCNTs/CPE	ITA	EGDMA	AIBN	Precipitation polymerization	SWASV	1.0 × 10^−11^–8.0 × 10^−8^ M	3.8 pM	Sea and river		[87]
	MWCNT-CH=CH_2_/Pt	AA	NNMBA	AIBN	Surface imprinting	DPV	1–5 ppm	2 × 10^−2^ μM	Waste water, lake, food sample and cosmetics		[92]
	MWCNTs/Fe_3_O_4_/C-PE	2-VP ^a^	EGDMA	AIBN	Surface imprinting	DPSV	3–55 μg/L	0.5 μg/L	River, waste water		[132]
	Fe_3_O_4_/GCE	4-VP	EGDMA	AIBN	Precipitation polymerization	DPV	0.1–80 ng/mL	0.05 ng/mL	Fruit juice, drinking water		[134,142]
Cd^2+^	MWCNT-CH=CH_2_/Pt	MAA	NNMBA	K_2_S_2_O_8_	Precipitation polymerization	DPV	1–5 ppm	0.03 μM	Lake water, pigments, cosmetics and fertilizers	3 ng/mL (Drin-king water), 3.0–5.0 μg/L (Human body)	[68,93]
	GO/GCE	Benzo[f]chrom-ene scaffold	EGDMA	AIBN	Thermal polymerization	ASV	4.2 × 10^−12^–5.6 × 10^−3^	7 × 10^−14^ M	Blood serum and human hair samples		[106]
	AuNPs/CPE	Thiosemicarba-mide functionalized CS; MAA	EGDMA	AIBN	Surface imprinting	DPV	10^−3^–100 μM	1.43 × 10^−10^ M	Rice, drinking water		[123]
	Fe_3_O_4_/SiO_2_/GCE	2-Aminobenzimi-dazole	EGDMA	AIBN	Precipitation polymerization	DPV	0.008–0.05 μM	1 × 10^−4^ μM	Waste and drinking water		[138]
Cu^2+^	GO/MWCNT/CPE	5-methyl-2-thiozylmethacryl-amide	EGDMA	AIBN	Precipitation polymerization	——	1.0 × 10^−6^–1.0 × 10^−1^ M	0.4 μM	Spiked river, dam, and tap water	31.5 μM (Dri-nking water)	[27]
	Fe_3_O_4_/C/GCE	NIPAM ^b^	MBA ^c^	APS	Surface imprinting	DPV	1.0 × 10^−5^–1.0 × 10^−3^ M	5.99 μM	River, tap, and mineral water		[135]
	Fe_3_O_4_/SiO_2_/CS/Naf-ion/GCE	CS	GA ^d^	——	Surface imprinting	DPASV	0.01–20 μM	5 nM	Tap and river water		[47]
Hg^2+^	MWCNTs/GCE	MAA	EGDMA	AIBN	Precipitation polymerization	DPV	0.01–700 μM	5.0 nM	Waste and ground water	2 μg/L (Drinking water), 0.249 μM (In-dustrial waste water), 0.05 mg/kg (Veg-etables)	[81]
	GO/IDE	Styrene	EGDMA	AIBN	Thermal polymerization	——	——	1 ppm	——		[105]
	RGO/GCE	MAA	EGDMA	APS	Surface imprinting	SWASV	0.07–80 μg/L	0.02 μg/L	Waste and drinking water		[109,143]
	Al_2_O_3_/rGO/CPE	MAA	EGDMA	AIBN	Thermal polymerization	——	0.004–1300 μM	0.00195 μM	Drinking water, industrial waste water, food and human hair		[110,144]
	GQDTU/GCE	GQDTU	EGDMA	AIBN	Suspension polymerisation	DPV	0.05–23 μM	23.5 nM	River and tap water		[114]
		GQDTU	EGDMA	AIBN	Suspension polymerisation	CV	0.06–0.85 and 1.4–7 μM	30.2 nM	River and tap water		[114]
	g-C_3_N_4_/CPE	ITA	EGDMA	AIBN	Precipitation polymerization	SWASV	0.06–25.0 nM	18 pM	Tap and sea water		[119]
	SCMNPs/CPE	Functional mercaptoethylam-ino monomer	EGDMA	APS	Self-assembly homo polymerization	SWV	0.20–1600.0 ng/mL	0.04 ng/mL	River, waste water, and vegetables samples		[139,142]

2-VP ^a^: 2-vinylpyridine; NIPAM ^b^: N-isopropyl acrylamide; MBA ^c^: N, N-methylene diacrylamide; GA ^d^: Glutaraldehyde.

## Data Availability

Not applicable.

## Abbreviations

HMIs	Heavy metal ions	MAA	Methacrylic acid
IIECSs	Ion-imprinted electrochemical sensors	OPD	O-phenylenediamine
IIPs	Ion-imprinted polymers	AA	Acrylic acid
WHO	World Health Organization	AM	Acrylamide
IQ	Intelligence quotient	4-VP	4-Vinylpyridine
SERS	Surface-enhanced Raman spectroscopy	Py	Pyrrole
AAS	Atomic absorption spectrometry	ECSs	Electrochemical sensors
CE	Capillary electrophoresis	CS	Chitosan
ICP-AES	Inductively coupled plasma-atomic emission spectroscopy	NNMBA	N,N′-methylene double acrylamide
IC-UV-vis	Ion chromatography-ultraviolet vis spectrometry	TPPZ	2,3,5,6-Tetra(2-pyridyl) pyrazine
ICP-MS	Inductively coupled plasma mass spectrometry	MMWCNTs	Magnetic multi-walled carbon nanotubes
MP	Microprobe	NPs	Nanoparticles
XFS	X-ray fluorescence spectroscopy	WE	Working electrodes
MIPs	Molecularly imprinted polymers	RE	Reference electrodes
MIECSs	Molecularly imprinted electrochemical sensors	CE	Counter electrodes
MIT	Molecular imprinting technique	ISEs	Ion selective electrodes
HMIIECSs	Heavy metal ions imprinted electrochemical sensors	LOD	Low limit of detection
CPE	Carbon paste electrode	IIMs	Ion-imprinted membranes
APA	Allyl phenoxyacetate	Th	Thiophene
EGDMA	Ethylene glycol dimethacrylate	SCE	Saturated calomel electrode
AIBN	Azobisisobutyronitrile	CV	Cyclic voltammetry
CNF	Carbon nanofibers-grafted	DPV	Differential pulse voltammetry
SEM	Scanning electron microscopy	PPD	P-phenylenediamine
AAM	Acryl amide	THPP	5,10,15,20-Tetrakis(3-hydroxyphenyl)-porphyrin
BAAP	Bis-(2-acryloylamino-ethyl)-phosphinic acid	IL	Ionic liquid
SPCE	Screen-printed carbon electrode	GR	Graphene
PGE	Pencil graphite electrode	rGO	Reduced graphene oxide
PC	Poly catechol	GP	Graphite powder
SPE	Screen printed electrode	EAAP	Ethyl 6-(allyloxy)-2-amino-4-phenyl-4H-benzo[f]chromene-3-carboxylate
DPC	1,5-Dipenylcarbazone	ASV	Anodic stripping voltammetry
CNTs	Carbon nanotubes	DDBHCT	2,2’-(9E,10E)-1,4-Dihydroxy-anthrace-9,10-dimethylene)bis(hydrazi-1-carbonthiona-mide)
SWCNTs	Single-walled carbon nanotubes	APS	Ammonium persulfate
GCE	Glassy carbon electrode	TEM	Transmission electron microscope
DPASV	Differential pulse anodic stripping voltammetry	PPy	Polypyrrole
PAN	1-(2-Pyridazo)-2-naphthol	ERGO	Electrochemical reduction of graphene oxide
LOQ	Limit of quantification	PoPD	Poly (o-phenylenediamine)
SWASV	Square wave anodic stripping voltammetry	EDTA	Ethylene diamine tetraacetic acid
RSD	Relative standard deviation	GQDs	Graphene quantum dots
EIS	Electrochemical impedance spectroscopy	GQDTU	Thiourea derivative-functionalized GQDs
LEDs	Light-emitting devices	g-C3N4	Graphitic carbonitride
ITA	Itaconic acid	NPG	Nanoporous gold
AuNPs	Gold nanomaterials	GE	Gold electrode
MMIECS	Magnetic molecularly imprinted electrochemical sensors	MNMs	Magnetic nanomaterials
GF-AAS	Graphite furnace atomic absorption spectrometry	2-CBT	Benzothiazole-2-carbaldehyde
APTES	3-Aminopropyltriethoxysilane	MPS	Methacryloxypropyltrimethoxysilane
VTES	Vinyltriethoxysilane	SCMNPs	SiO_2_-coated magnetite nanomaterials
PVC	Polyvinylchloride		
